# Novel hexameric tin carboxylate clusters as efficient negative-tone EUV photoresists: high resolution with well-defined patterns under low energy doses†

**DOI:** 10.1039/d3na00131h

**Published:** 2023-04-28

**Authors:** Jia-Rong Wu, Ting-An Lin, Yan-Ru Wu, Po-Hsiung Chen, Tsi-Sheng Gau, Burn-Jeng Lin, Po-Wen Chiu, Rai-Shung Liu

**Affiliations:** a Frontier Research Center for Matter Science and Technology, Department of Chemistry, National Tsing-Hua University Hsinchu Taiwan 30013 ROC rsliu@mx.nthu.edu.tw; b TSMC-NTHU Joint Research Center, National Tsing-Hua University Hsinchu Taiwan 30013 ROC burnlin@ee.nthu.edu.tw tsaisheng_kao@mx.nthu.edu.tw; c College of Semiconductor Research ROC; d Department of Electrical Engineering, National Tsing-Hua University Hsinchu Taiwan ROC rsliu@mx.nthu.edu.tw pwchiu@ee.nthu.edu.tw

## Abstract

Synthesis of two novel tin carboxylate clusters (RSn)_6_(R′CO_2_)_8_O_4_Cl_2_ is described, and their structures have been characterized by X-ray diffraction. These clusters have irregular ladder geometry to form very smooth films with small surface roughness (RMS <0.7 nm) over a large domain. EUV lithography can be used to resolve half pitches (HPs) in the order of 15–16 nm with line width roughness (LWR = 4.5–6.0 nm) using small doses (20–90 mJ cm^−2^). Cluster 1 (R = *n*-butyl; R′CO_2_ = 2-methyl-3-butenoate) contains only a radical precursor and cluster 2 (R = vinyl, R′CO_2_ = 2-methylbutyrate) bears both a radical precursor and an acceptor; the latter is much better than the former in EUV and e-beam photosensitivity. For these clusters, the mechanisms of EUV irradiation have been elucidated with high resolution X-ray photoelectron spectroscopy (HRXPS) and reflective Fourier-transform infrared spectroscopy (FTIR). At low EUV doses, two clusters undergo a Sn–Cl bond cleavage together with a typical decarboxylation to generate carbon radicals. The *n*-butyl groups of cluster 1 are prone to cleavage whereas the vinyl–Sn bonds of species 2 are inert toward EUV irradiation; participation of radical polymerization is evident for the latter.

## Abstract 2

Synthesis of two novel tin carboxylate clusters (RSn)_6_(R′CO_2_)_8_O_4_Cl_2_ is described, and their structures have been characterized by X-ray diffraction.

## Graphical abstract

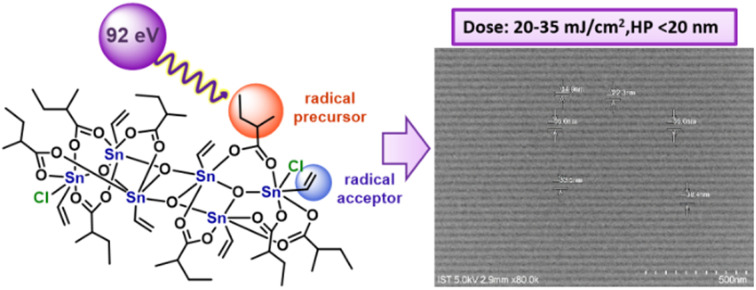

## Introduction

**Scheme 1:**
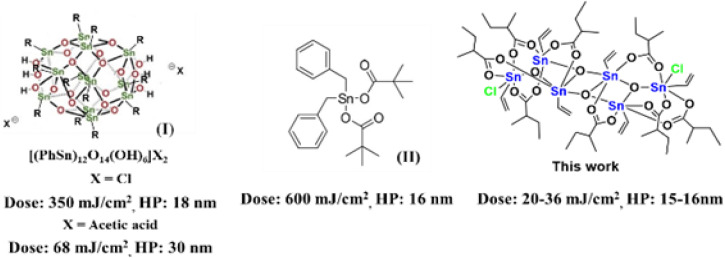
Tin-derived photoresists and their EUV photosensitivity.

Inorganic complexes are promising photoresist materials in EUV (extreme ultraviolet) lithography.^1–4^ One obvious advantage is that the EUV absorption efficiencies of metal complexes such as Hf,^5–12^ Zr,^13–18^ Zn,^10,19,20^ and Sn^21–25^ are 2–3 folds as large as those of polymer-based photoresists. Despite intensive studies, very few inorganic photoresists^3,19^ can reach high resolution EUV patterns with half pitches (HPs) <20 nm under low dosage <50 mJ cm^−2^. Sn-based photoresists^21–25^ are important EUV materials because of their low cost and strong EUV absorption property. Inorganic tin-based photoresists have been developed mainly with 12-tin oxide clusters (I)^21,23–25^ and mononuclear tin complexes (II),^22^ which can produce EUV patterns with 16–18 nm HP, albeit with energies >300 mJ cm^−2^ (see Scheme 1). Tin carboxylate clusters are readily synthesized in several families,^26–28^ and their EUV applications have never been explored. One fear with metal carboxylate clusters as photoresists is the facile decomposition of carboxylate ligands by EUV light, leading to a large shrinkage of film thickness. This problem can be circumvented with new molecular design to allow one or two carboxylate ligands for decomposition. This work reports the synthesis of two new tin clusters (RSn)_6_Cl_2_O_4_(R′CO_2_)_8_, which have ladder-type structures. Most inorganic photoresists are prepared in sphere, ball or drum shapes; our ladder structures are somewhat irregular and tend to form smooth surface planes. The two chloro ligands are smaller than carboxylates in size, further minimizing the intermolecular distance. These clusters serve as negative-tone EUV photoresists to produce EUV lithographic patterns with a resolution up to 15–16 nm HP with only 20–36 mJ cm^−2^ for the vinyltin-containing cluster (2). Notably, cluster 2 bears both radical precursors such as carboxylate ligands and radical acceptors such as vinyltin. For the other cluster 1 containing Sn–Bu moieties, the line/edge/space character is reasonable with *L*/*S* = 1 and line width roughness (LWR) *ca.* 4.5–6.0 nm although high EUV doses of 80–90 mJ cm^−2^ are required to achieve high resolution patterns. XPS and FT-IR studies have been conducted to get insights of the EUV photolytic chemistry of one representative 2, in which carboxylate ligands are decomposed by EUV to release CO_2_ and isobutyl radicals, further facilitating the radical polymerization of the vinyltin moiety. Such a bifunctional nature as in cluster 2 proves to be a viable design to obtain outstanding negative-tone EUV photoresists.

## Results and discussion

**Scheme 2:**
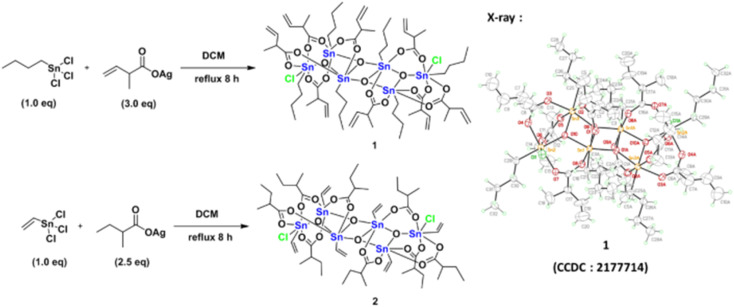
Synthesis of two photoresists.

The reaction of PhSnCl_3_ and RCO_2_Ag (>3.0 equiv.) was reported to form a chloride-free ladder cluster (PhSn)_6_(RCO_2_)_10_O_4_.^26^ As shown in Scheme 2, the reaction of BuSnCl_3_ with silver 2-methyl-3-butenoate (3.0 equiv.) delivered a new hexameric tin cluster (BuSn)_6_(C_4_H_7_CO_2_)_8_O_4_Cl_2_, which is designated as cluster 1; its X-ray structure has been determined to reveal a ladder structure containing two Sn–Cl bonds and eight carboxylate ligands in a (BuSn)_6_(C_4_H_7_CO_2_)_8_O_4_Cl_2_ cluster. The crystallographic data of cluster 2 have been deposited by us at the Cambridge X-ray Deposition Center (CCDC 2177714). Cluster 1 might have the following advantages as EUV photoresists: (i) a weak Sn–Cl bond can undergo EUV-driven cleavage to enhance molecular aggregation (ii) less shrinkage in film thickness is expected for compound 1 because two carboxylate ligands are absent. Cluster 2 was prepared similarly from the reaction of vinylSnCl_3_ and silver 2-methylbutyrate (2.5 equiv.). The vinyltin moiety in cluster 2 can accept a carbon radical to enhance the polymerization, which is a general approach to minimize EUV doses for a negative tone photoresist.^1–4^ The molecular structure of cluster 2 has also been determined by X-ray diffraction (CCDC 2184478).

**Fig. 1:**
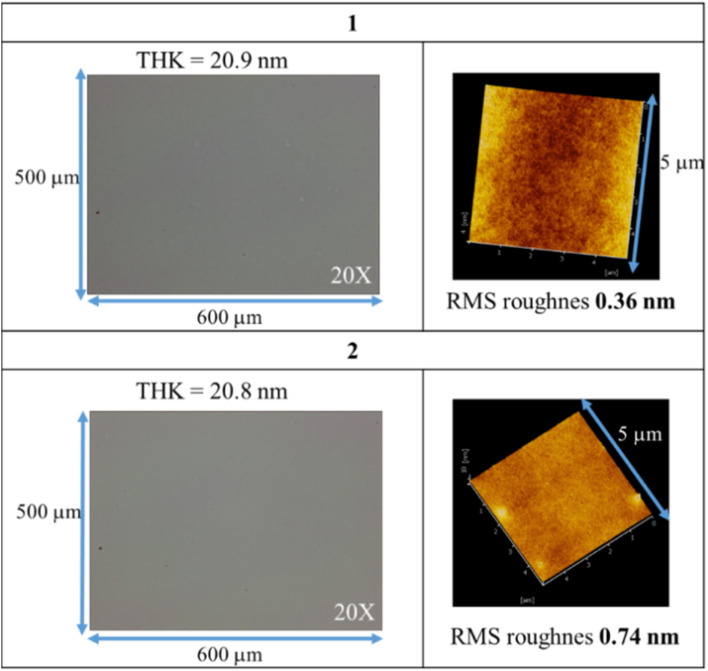
OM and AFM images of thin films after post application baking at 70 °C and 60 °C for 60 s, respectively for clusters 1 and 2.

Clusters 1 and 2 have a ladder geometry that has a large surface area; the two chloride ligands render the structures to become irregular. This structural feature is expected to resist the formation of a microcrystalline solid when the solution is concentrated on spin casting. Fig. 1 shows that the optical microscopy (OM) and atomic force microscopy (AFM) images of the thin films from a spin coating of the 1.5 wt% solution in 4-methyl-2-pentanol; the PAB (post application bake) is performed at 70 °C and 60 °C for 60 s, respectively for clusters 1 and 2. The thickness is 20.9 and 20.8 nm for clusters 1 and 2 respectively. No visible defects were observed over a large domain 0.5 × 0.6 mm according to the OM image. More importantly, AFM images over a 5 × 5 μm domain indicates a very smooth plane; their RMS roughness is only 0.36 and 0.74 nm for clusters 1 and 2 respectively. Highly smooth surfaces are required to minimize surface defects after EUV exposure. In contrast, a ball-like 12-tin oxide cluster I (X = OH, Scheme 1) is reported to have RMS roughness >3 nm.^29^

**Fig. 2:**
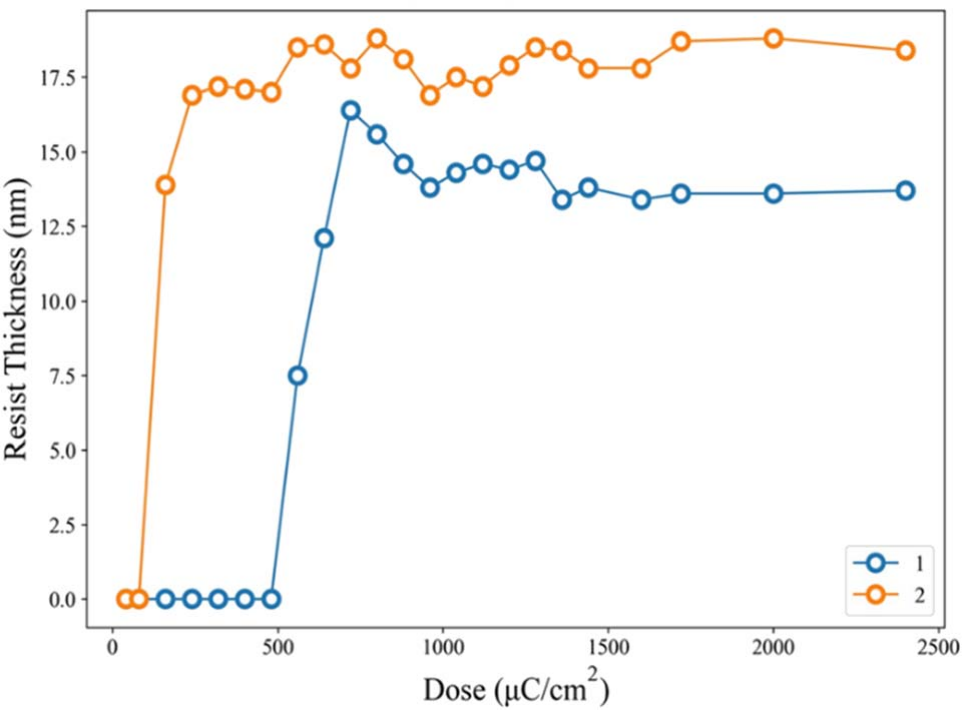
Contrast curves using e-beam as energy doses. Initial thickness for 1 (20 nm), 2 (24 nm) and post application baking at 70 °C and 60 °C for 60 s, respectively for clusters 1 and 2.

Fig. 2 shows the e-beam exposure contrast curves, which show the thickness of the exposed resist not removed by the developer; a function of thickness *versus* e-beam doses is plotted to show a typical negative-tone pattern. The initial thickness for clusters 1 and 2 is 20 nm and 24 nm respectively. Cluster 1 is less photosensitive than cluster 2 as their respective maximum is at 720 μC cm^−2^ and 240 μC cm^−2^ respectively. The superior sensitivity of cluster 2 is due to the role of the radical acceptor vinyltin, whereas its 2-methylbutyrate ligand is readily decomposed by EUV to generate the 2-methylbutyl radical. At 1000 μC cm^−2^, clusters 1 and 2 lose a large proportion in the film thickness, *ca.* 38% and 34% respectively. The energy of electrons is so high that a large portion of organic groups can be eliminated at high e-beam doses.

**Fig. 3:**
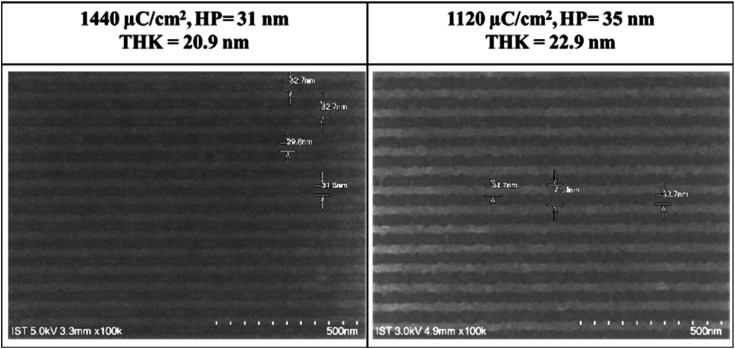
SEM images of e-beam lithography of clusters 1 (left) and 2 (right); PEB: 80 °C, 60 s; the scale bar corresponds to 50 × 10 nm.

Fig. 3 shows the SEM images (100 K) of two lithographic patterns for clusters 1 and 2 using e-beam as the energy source; the exposed films were baked at 80 °C for 60 s before the cleaning of the unexposed photoresist with 2-heptanone (60 s). E-beam lithographic patterns of HP = 31 and 35 nm were obtained with 1440 μC cm^−2^ and 1120 μC cm^−2^. Attempts to obtain small HP <30 nm patterns are unsuccessful due to overexposure.

**Fig. 4:**
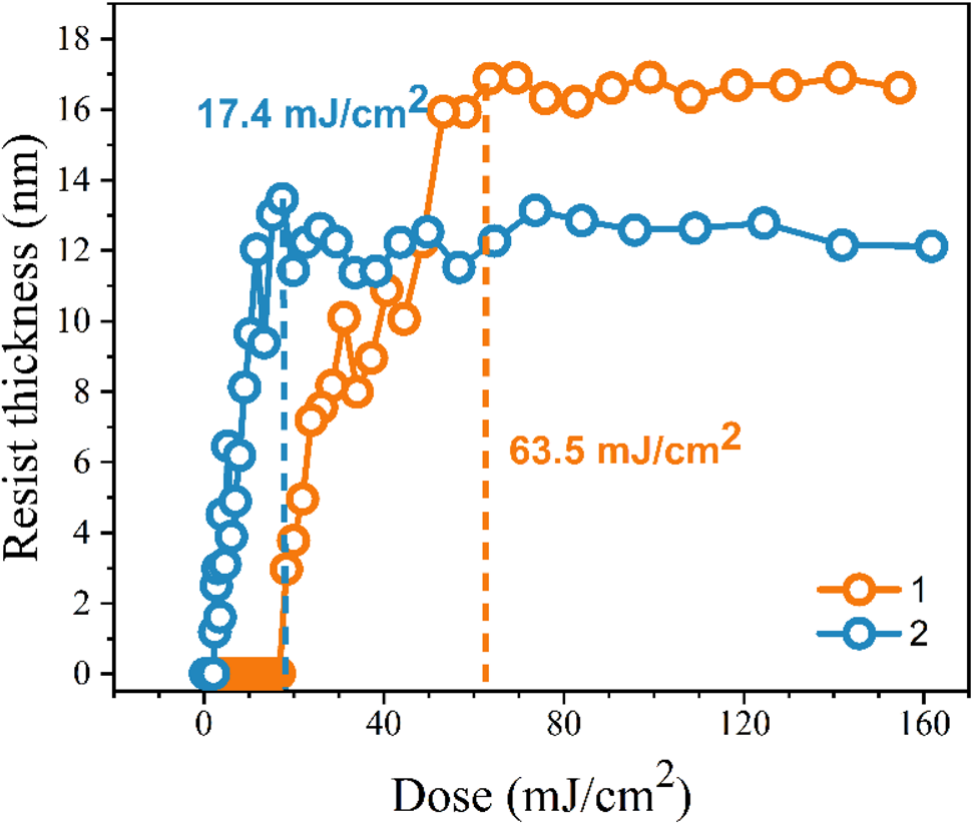
Contrast curve of tin-oxide photoresist 1 (orange) and 2 (blue) using EUV as energy doses; initial thickness 24 nm.

Our next task is to achieve lithographic patterns using EUV as the energy source. The EUV-exposure service was provided by the commercial slots of Swiss Paul Scherrer Institute Center (PSI) with EUV light at 13.5 nm. The pattern in Fig. 4 is typical of a negative-tone photoresist. The EUV doses start from 5.8 mJ cm^−2^ at an increment of 3–5 mJ cm^−2^. Cluster 2 turns out to be more photosensitive than cluster 1 because the former takes less energy to develop the EUV pattern. Small energy is achieved at 17.4 mJ cm^−2^ for cluster 2 to reach the maximum; the height remains constant from 17.4 to 160 mJ cm^−2^. In contrast, cluster 1 requires a high dose of energy (63.5 mJ cm^−2^) to achieve a mature pattern. The superior photosensitivity of cluster 2 is reflected by both EUV and e-beam lithography.

**Fig. 5:**
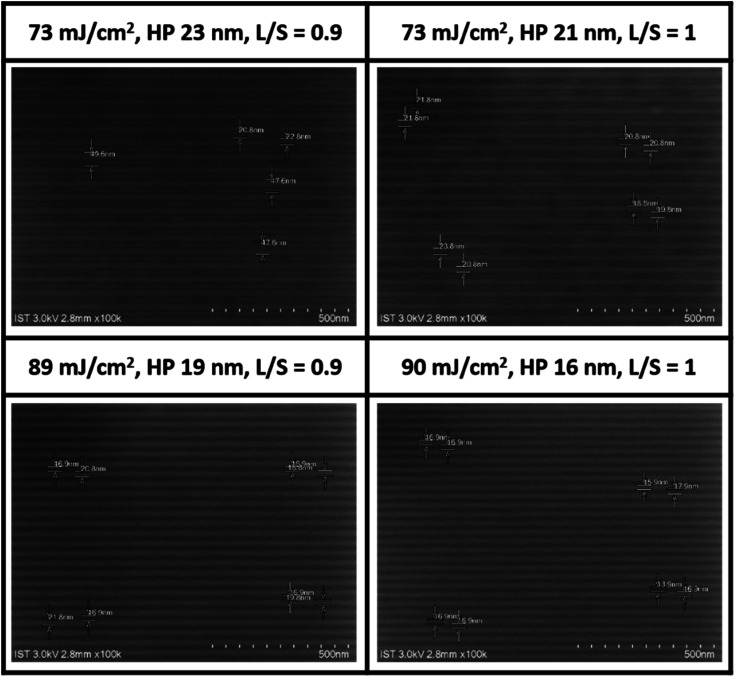
SEM images (100 K) of clusters 1 after EUV development, THK = 20.9 nm, PEB: 80 °C (60 s), 2-heptanone, 60 s, photoresist (white).A scale bar represents 50 × 10 nm.

The EUV lithographic pattern is further developed for cluster 1 under the same processing conditions as in the e-beam study, involving PAB (70 °C, 60 s), PEB (post-exposure bake, 80 °C, 60 s) and 2-heptanone as the developer (60 s). The EUV exposures were performed at the Swiss PSI center. Fig. 5 shows four selected SEM images of small HP patterns (16–23 nm) for clusters 1. Remaining SEM images of different HP patterns (16–50 nm HP) and other energy doses (50–100 mJ cm^−2^) are provided in the ESI.† To our pleasure, the patterns can be resolved into small HP including 23, 21, 19 and 16 nm. The energy doses are 73 mJ cm^−2^ for HP 21 and 23 nm, and 89–90 mJ cm^−2^ for HP 19 and 16 nm; notably standard line/space (*L*/*S*) = 1.0–1.1 patterns still remain even for the 16 nm HP pattern. In small half pitch patterns, most photoresists have blurring, tangling and breaking problems, which are less stringent in our cases. In Fig. 5 (bottom), the HP = 18–19 nm patterns are calculated to have line width roughness (LWR) *ca.* 4.6–4.9 nm (see Table S1, ESI†), again showing reasonable line/space/edge characters.

**Fig. 6:**
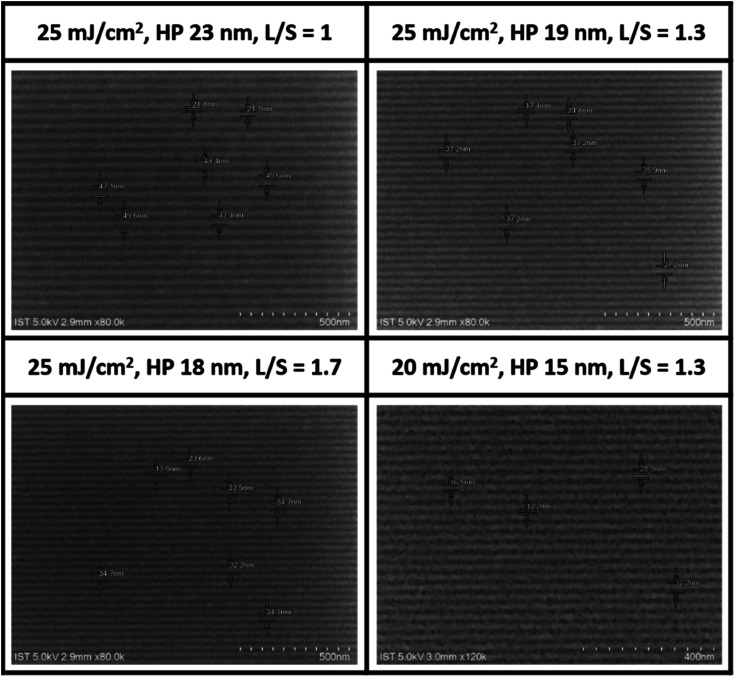
SEM images of clusters 2 after EUV development, THK 22.9 nm, 2-heptanone, 60 s, no PEB, photoresist (white). A scale bar represents 50 × 10 nm (top three) and 40 × 10 nm (bottom).

EUV lithographic patterns for cluster 2 show strong photosensitivity. No PEB is necessary after EUV exposure because cluster 2 tends to show photoresist blurring at small pitches. Fig. 6 shows the successful development of small HP lithography including 23, 18, 17 and 15 nm patterns; the dose energies are as low as 20–25 mJ cm^−2^. SEM images of other patterns (13–52 nm HP) at different EUV doses (16–52 mJ cm^−2^) are provided in the ESI.† Small half pitches and low energy doses highlight the value of cluster 2 as a EUV photoresist.

Our next task is to examine the quality of line/space/edge characters, and the corresponding line/space (*L*/*S*) = 1.0, 1.3 and 1.7 are respectively obtained for HP = 23, 19 and 18 nm all at 25 mJ cm^−2^. Consistently increasing *L*/*S* values for a smaller HP pattern is presumably due to a rapidly radical polymerization at the vinyltin moiety to increase the blurring. Although no photoresist tangling or breaking was present here, significant photoresist blurring occurred with the 15 nm HP pattern, rendering the *L*/*S* estimation to become inaccurate. In the case of the 23 nm HP pattern at 35 mJ cm^−2^, the corresponding line width roughness (LWR) is calculated to be 5.7 nm using suitable software.^30^

**Fig. 7:**
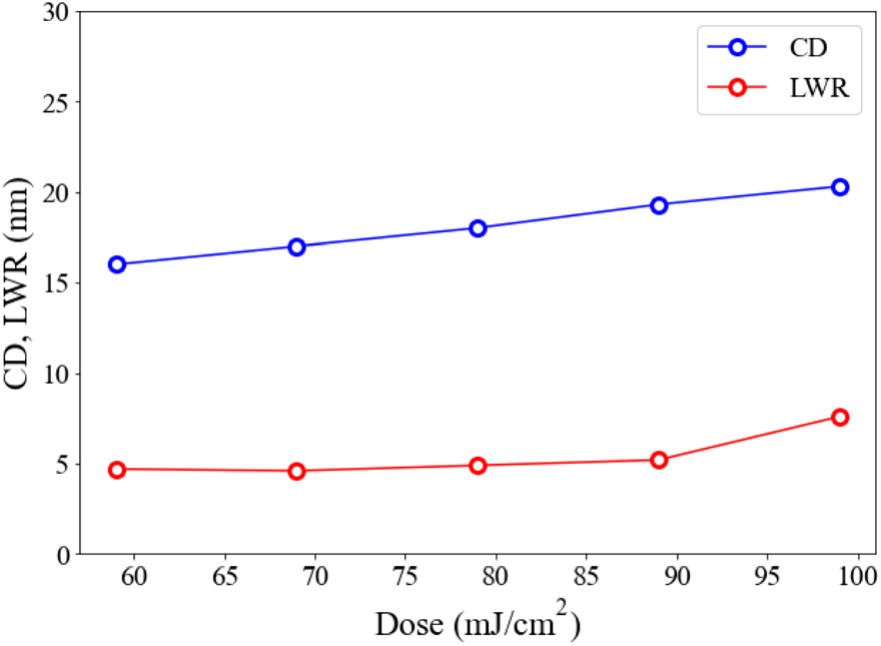
A plot of LWR and CD *versus* doses for cluster 1 at HP = 18 nm.

Cluster 1 is better than the other cluster 2 in EUV pattern quality; the former can provide a number of reliable measurable LWR (line width roughness) and CD (critical dimension) at different doses. Fig. 7 shows a plot of such variables at small 19 nm HP. LWR values are kept nearly constant at 4.6–4.9 nm from *J* = 56 to 89 mJ cm^−2^, but it increased abruptly at *J* = 99 mJ cm^−2^. This range reflects good control of line/space quality. To measure the photoresist blurring, we note a slow increase of the critical dimension of the photoresist from a value of 15.5 nm to 19.5 nm over this entire dose region. The CD increase is a general problem that hinders achieving small HP pitch patterns.

**Fig. 8:**
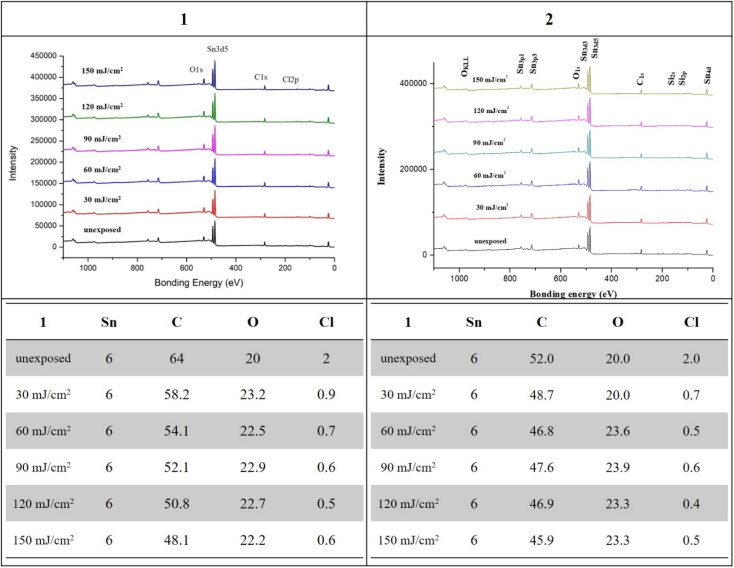
XPS analysis at different doses, species 1 (left) and 2 (right).

XPS was employed to monitor the chemical composition of exposed thin films at different EUV doses; the results are shown in Fig. 8. The EUV exposed films are subject to pattern development to locate the exposed area. For cluster 1, a loss of 5.8 carbon and 1.1 chloride atoms was obtained at 30 mJ cm^−2^, but strangely 3.2 oxygen atoms are increased. This oxygen increase becomes clear after FTIR study indicates a cleavage of the Sn–X bonds (X = Cl and *n*-butyl) to form the corresponding Sn–OH bonds in our EUV radiation/air exposure procedure. With increasing doses from 50 to 150 mJ cm^−2^, a slow and gradual loss of carbon and chloride atoms is observed, but the oxygen content remains unchanged. As the pattern is developed at 90 mJ cm^−2^, there are 12 carbons disappearing while oxygen levels remain unchanged. For species 1, such a significant carbon loss is incompatible with a radical polymerization. Photoresist 2 also follows the same XPS pattern in which only 3.3 carbons and 1.3 chloride atoms are lost, while additional 3.6 oxygen atoms are present at 30 mJ cm^−2^ at which a EUV pattern is well developed. Again, we observed a small loss of carbon and chloride contents as the doses are increased from *J* = 60 to 150 mJ cm^−2^ whereas the oxygen content remains unchanged. The small loss of carboxylate ligands for species 2 is due to a radical polymerization with vinyltin as the radical acceptor, which is further supported by high resolution XPS analysis and FT-IR study.

**Fig. 9:**
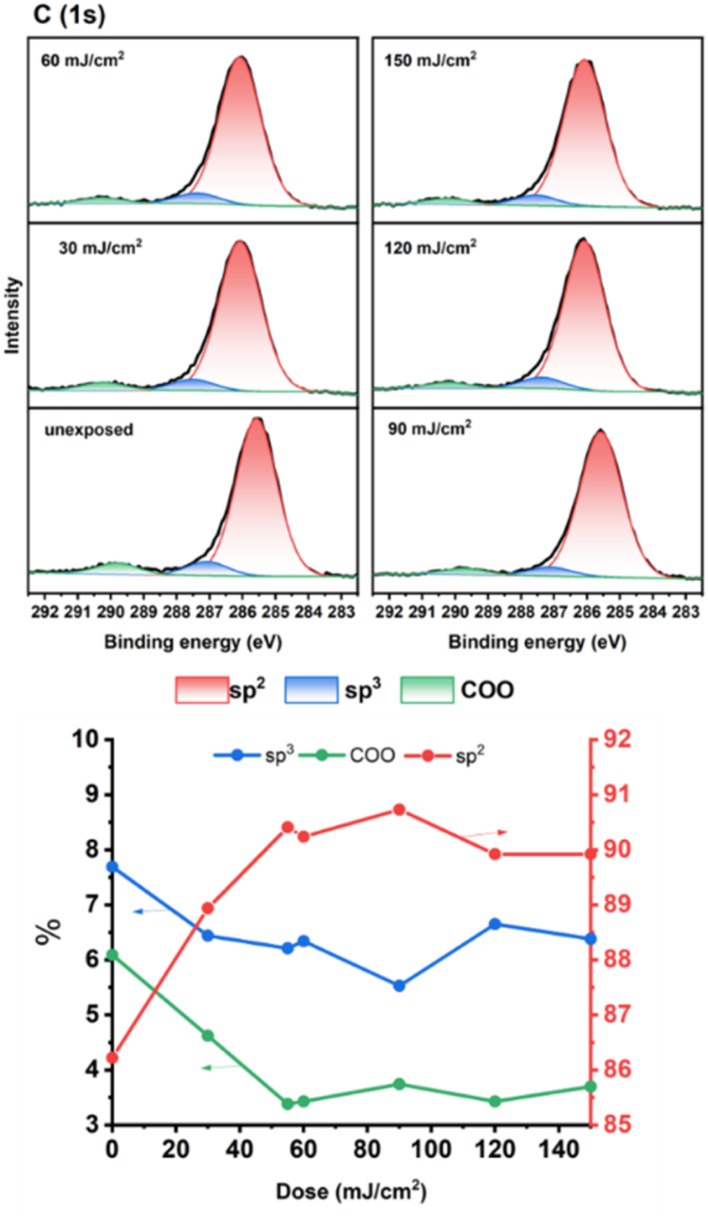
HRXPS analysis of photoresist 2 on C(1s).

**Fig. 10:**
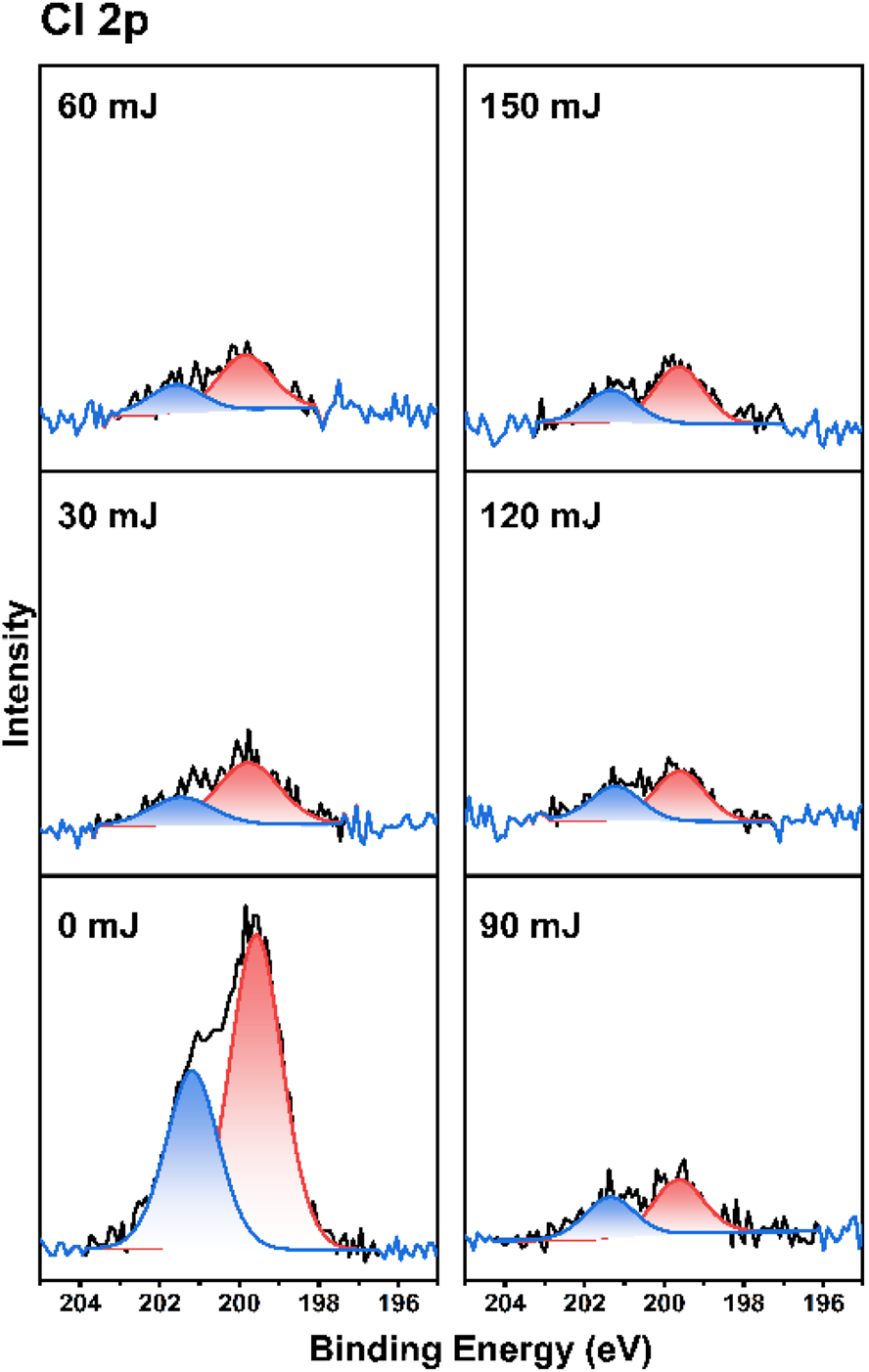
HRXPS analysis for the chloro ligands.

As cluster 2 is more efficient than species 1 in EUV photosensitivity, high resolution XPS (HRXPS) analysis was conducted to examine the C(1s) absorption bands (see Fig. 9). There are three components corresponding to the sp^3^- and sp^2^-hydridized carbons together with the CO_2_-carbons, centered at 285.58, 287.00 and 289.72 respectively.^31^ After EUV irradiation from 30 to 150 mJ cm^−2^, a gradual 4–6% increase in the sp^2^-carbon contents is observed whereas the sp^3^-carbon loses 15–28% contents, and CO_2_-loss is up to 25–48%. This information indicates that decarboxylation reactions are the dominant pathway, but few vinyl tin groups can undergo radical polymerization to avoid a large loss of sp^3^-carbons. This assessment is also supported by FT-IR spectra. The analysis of high resolution XPS associated with Cl(2P) absorption bands is also performed. Two bands at 199.64 and 201.33 eV are assigned to the 2P(1/2) and 2P(3/2) absorption bands of the Sn–Cl moieties of photoresist 2. We note that the two bands remained the same even at 30–150 mJ cm^−2^ although the intensity becomes weak (Fig. 10).

**Fig. 11:**
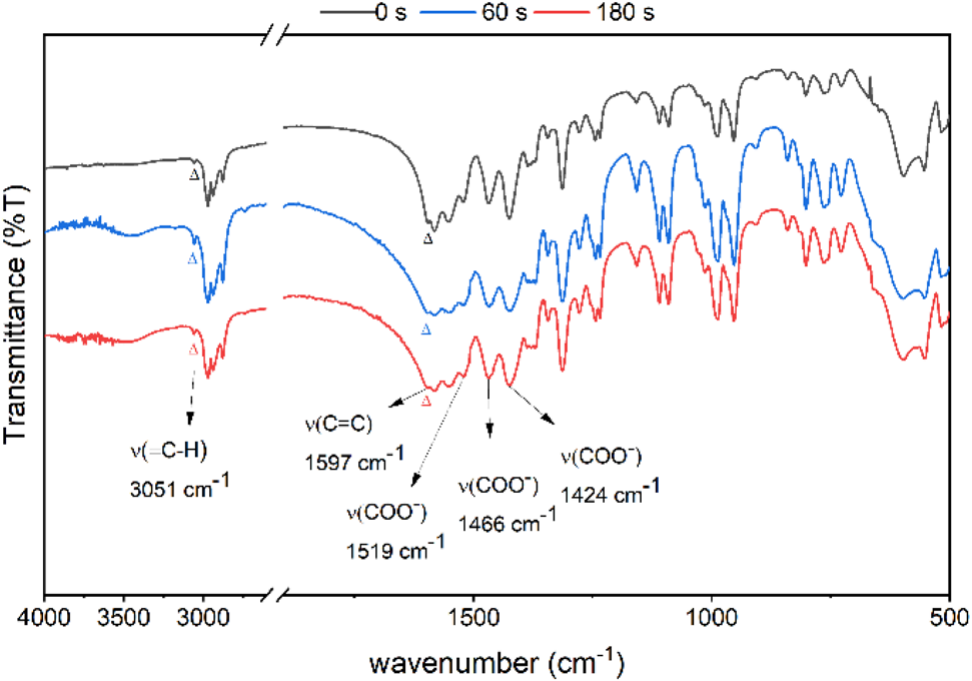
FT-IR of cluster 2 in KBr (a) after vacuo, 1 min (top) (b) heating at 80 °C for 60 s (middle), and (c) 80 °C for 180 s (bottom).

Reflective FT-IR spectra were used to examine the thermal chemistry of photoresist 2 in KBr pellets; see Fig. 11. Cluster 2 was placed *in vacuo* for 1 min before KBr preparation. The sample was placed in a glass vessel at 80 °C for 1 and 3 min before making KBr pellets. The vinyltin bonds of cluster 2 might be removed using water in the presence of a Brønsted acid. On heating the sample in 80 °C for 60 and 180 s, the two KBr pellets show identical spectra to that of the authentic sample. One important feature is the observation of a weak *ν*(=C–H) band at 3051 cm^−1^ and a *ν*(C=C) band at 1597 cm^−1^,^32^ which match well those (3031 and 1580 cm^−1^) of Sn(CH=CH_2_)_4_ (see Fig. S8†).^32^ Photoresist 2 is very stable at 80 °C on brief heating.

**Fig. 12:**
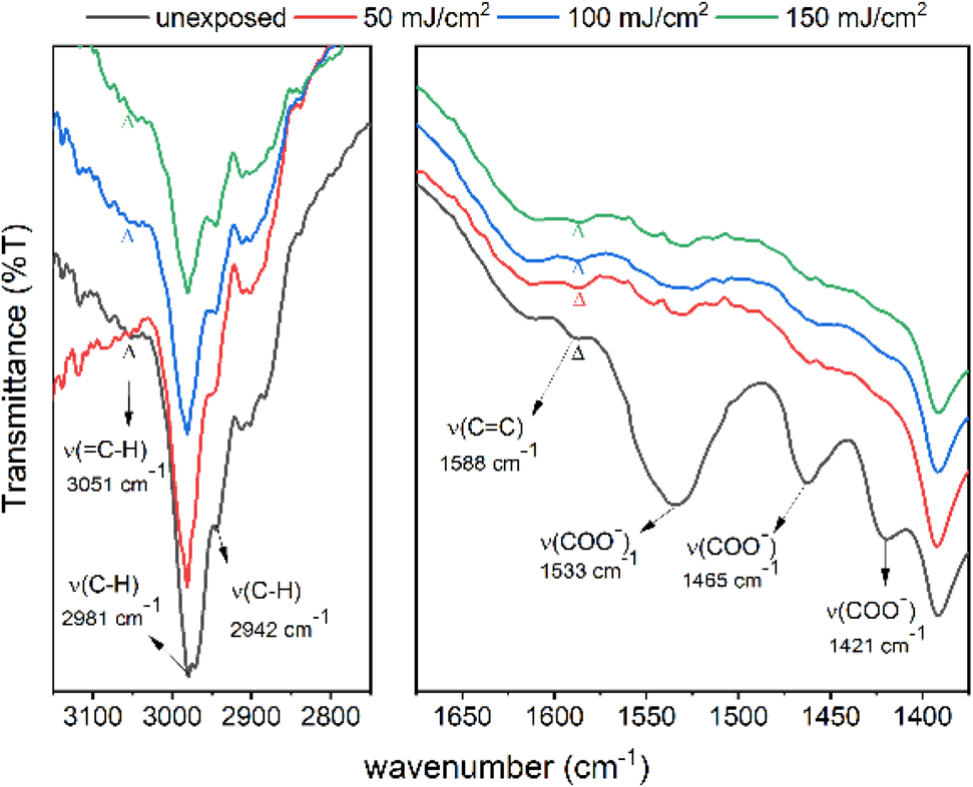
FT-IR of the thin films of cluster 2 for the unexposed film, 50 mJ cm^−2^, 100 mJ cm^−2^ and 150 mJ cm^−2^ (from bottom to top). The left spectra are enlarged in size by three fold to show the clarity.

The effects of EUV doses were studied on cluster 2 with a thick film (>1 μm) coated on a Si wafer. The EUV doses are applied with *ca.* 75, 150 and 200 mJ cm^−2^. Pattern development is required to find the exposed area. As shown in Fig. 12, we are able to identify the stretching bands of C–H, C=C, and O–C=O on the unexposed film. On these refractive IR spectra, the *ν*(=C–H) band is located at 3051 cm^−1^ as a small bump whereas the *ν*(C=C) band is clearly shown at 1588 cm^−1^. The 1533 cm^−1^ band is assigned to an asymmetric stretching mode of tin carboxylate groups together with two 1466 and 1421 cm^−1^ bands assignable to the symmetric stretching modes. After exposure to 50, 100 and 150 mJ cm^−2^, the three carboxylate absorptions (1425–1520 cm^−1^) lose intensity very quickly whereas the *ν*(C=C) band intensity remains little changed at 50 mJ cm^−2^. However, the *ν*(C=C) absorption intensity gradually decreases at high EUV doses of 100 and 150 mJ cm^−2^. Similar to HRXPS studies, tin carboxylate ligands are much more degradable than vinyltin ligands in EUV photolytic decomposition. As shown in the whole spectra (see Fig. S9†), carboxylate vibration intensity (1530–1420 cm^−1^) disappears much more rapidly than that of the alkyl C–H bonds (2850–3000 cm^−1^). Evidently, small proportions of vinyltin groups participate in radical polymerization to avoid a loss of the sp^3^-hybridized *ν*(C–H) absorption intensity.

**Fig. 13:**
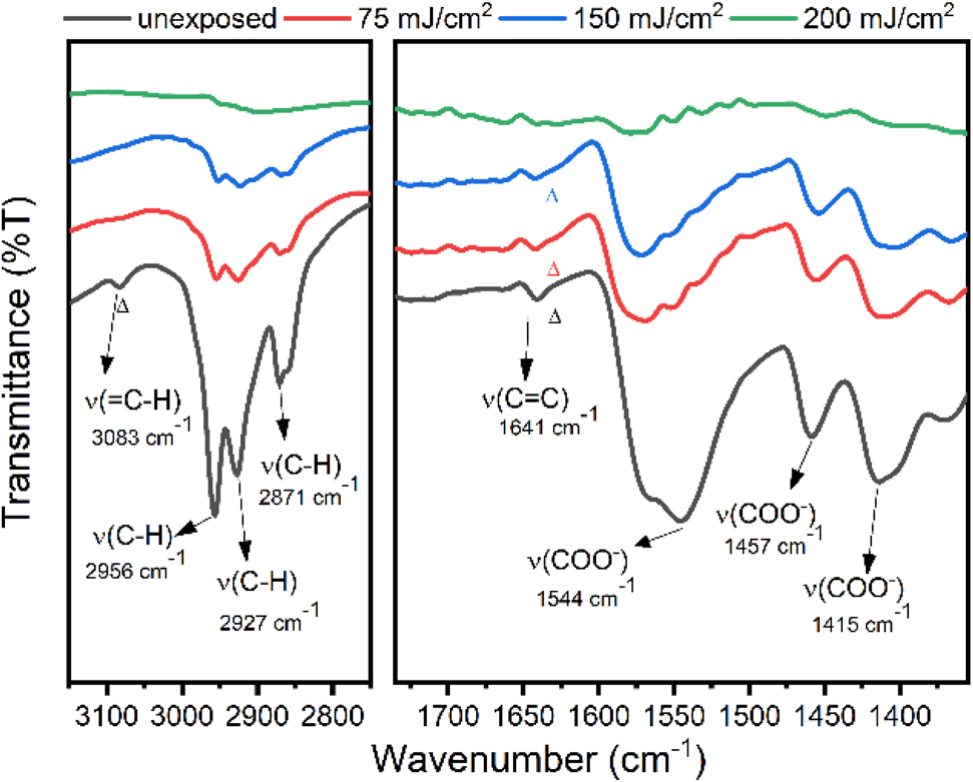
FT-IR of exposed film for species 1.

Reflective FT-IR spectra were investigated to study the aggregation mechanism of photoresist 1 with EUV doses 75, 150 and 200 mJ cm^−2^. As shown in Fig. 13, after exposure to 75 and 150 mJ cm^−2^, the aliphatic *ν*(C–H) and vinyl *ν*(=C–H) bands in the 2800–3100 cm^−1^ region lose intensity as rapidly as those of carboxylate ligands (1550–1440 cm^−1^), showing facile decomposition of not only carboxylate ligands but also the Sn–butyl bonds; Sn–butyl is weaker than vinyltin in terms of the Sn–C bond strength. Photolytic cleavage of a Sn–Bu bond is well established on 12-tin oxide clusters.^33,34^ These refractive FTIR spectra show complete decomposition of tin ligands with no polymerization of the 2-methyl-3-butenoate ligands for photoresist 1.

**Scheme 3:**
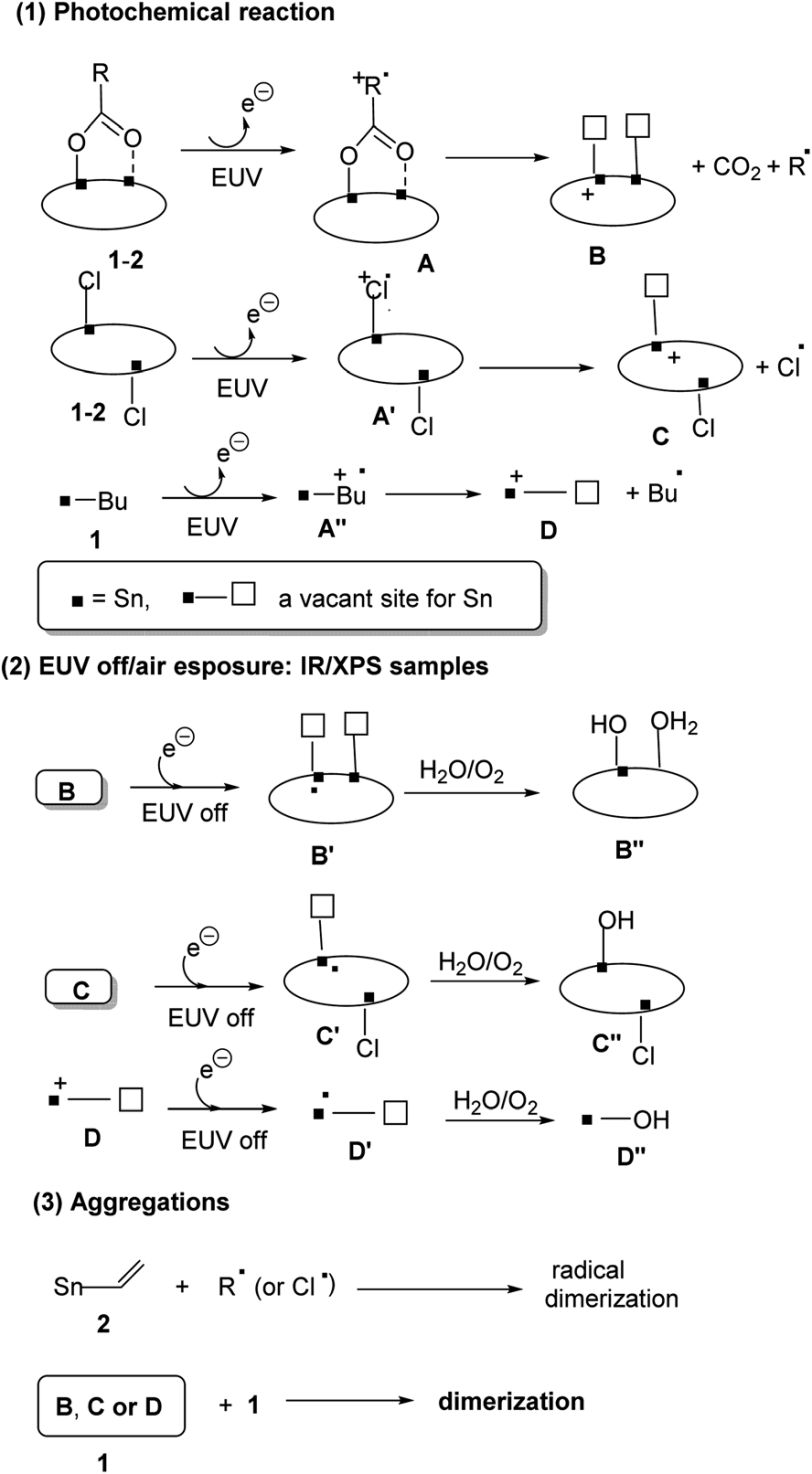
Proposed EUV reaction mechanism.

A mechanism is postulated in Scheme 3 to rationalize the observation of HRXPS and FT-IR data in Fig. 9–12. As EUV was operated in a high vacuum system, the amount of water on the film is very limited. With EUV excitation, clusters 1 and 2 undergo photolytic ionization to form species A and A′ to release an electron,^33,34^ further yielding two cations B and C together with CO_2_ and Cl and R radicals. Cluster 1 releases an additional *n*-butyl radical with a facile Sn–butyl bond cleavage. When the EUV light is off, recombination of free electrons with radical cation species B, C and D yielded reactive Sn(iii) intermediates B′, C′ and D′, which are further oxidized by O_2_/H_2_O after air exposure, yielding Sn–OH containing species B′′, C′′ and D′′. This proposed mechanism well explains our XPS study at *J* = 30–60 mJ cm^−2^ that oxygen content is increased with the elimination of carboxylate, Sn-butyl and chloro ligands for tin cluster 1, further forming new Sn–O bonds. In the case of photoresist 2, molecular aggregation relies on radical polymerization of its vinyltin moiety; this process is clearly indicated by refractive FTIR and HRXPS analysis. For cluster 1, the aggregation follows a well-known process involving the combination of intermediates B, C and D with a second molecule 1 as FTIR spectra showed rapid decomposition of both Sn-butyl and Sn-carboxylate fragments whereas a polymerization process is not supported at all.

## Conclusions

Organic tin compounds are cheap and potentially useful as EUV photoresists; very few metal-based photoresists can resolve small half-pitch patterns (<20 nm) with low EUV doses (<50 mJ cm^−2^). A breakthrough is made *via* chemical synthesis of two ladder-type carboxylate clusters 1 and 2. Because of irregular geometry, the two clusters form very smooth surfaces with small surface roughness (<0.8 nm) over a large domain according to OM and AFM characterization. Although the two clusters are unsatisfactory in e-beam lithographic development, the performance in EUV lithography is astonishing because of well-defined patterns in small half-pitch lithography, 15–16 nm HP, *J* = 20–80 mJ cm^−2^; no surface defects are present with regard to the blurring, crosslinking and breaking. HRXPS and reflective IR studies reveal that both clusters undergo facile Sn–Cl and Sn–carboxylate decomposition. Under EUV radiation, the vinyltin moiety of photoresist 2 is relatively inert whereas the butyltin fragment of cluster 1 is readily cleaved. Polymerization occurs clearly on cluster 2, but cluster 1 undergoes decomposition of all tin ligands.

## Experimental section

### Material preparation and characterization

All chemicals were purchased from the commercial supplier Sigma. ^1^H NMR and ^13^C NMR spectra were recorded on a Bruker 400 MHz, or Bruker 500 MHz spectrometer using chloroform-d (CDCl_3_) as the internal standard. TGA was performed using a Mettler-Toledo 2-HT at the heating rate of 8 °C min^−1^. FTIR spectroscopy of powder samples was conducted using a Bruker Vertex 80v spectrometer.

### Thin-film deposition

Cluster 1 or 2, at 1.5–1.75 wt% was dissolved in 4-methyl-2-pentanol; the solution was filtered through a 0.22 μm filter. The resist film was deposited by spin-coating at 1200 rpm for 10 s and 1600 rpm for 25 s on a SiO_2_-coated (THK 100 nm) Si-wafer. The wafer was baked at 70 °C and 60 °C for 60 s, respectively for clusters 1 and 2. The thickness of the thin films was in the range of 20.9–22.9 nm, which was measured by using a J. A. Woollam M2000. Atomic force microscopy (AFM) images were measured with a SEIKO SPA-300 HV, using the contact mode. These films were also used for e-beam and EUV exposure.

### Electron-beam lithography (EBL)

Electron-beam lithography was performed on an Elionix ELS-7800 with an accelerating voltage of 80 kV, beam current of 200 pA for the contrast curve and 50 pA for the line pattern. After exposure, the samples were developed with 2-heptanone for 60 s and rinsed with deionized water. To evaluate the contrast curve of the photoresist, a series of squares (50 × 50 mm^2^) were prepared, and each with different dosages varied from 80 μC cm^−2^ to 2400 μC cm^−2^. The contrast curve was then obtained by measuring the remaining thickness of each exposed square area through an *α*-step tool after solvent development. To analyze the resolution limit of our synthesized resists, different dense line features are designed from 50 nm HP down to 20 nm HP, and the exposure dosage to obtain the best resolution pattern was optimized at 1440 and 1120 μC cm^−2^, respectively for clusters 1 and 2.

### EUV-IL exposure and LWR measurement

Periodic aerial images were generated by two-beam interference in an EUV interference lithography (EUV-IL) system. The EUV-IL exposure at the Swiss Light Sources (SLS), Paul Scherrer Institute, utilizes 13.5 nm light with a high spatial coherence length and uniform illumination, to transmit a grating mask. This mask consists of multiple grating pairs with periods ranging from 100 nm HP to 32 nm HP, and the period of the first-order interference on the resist is half of that on the mask grating. Dosage on the mask (Dose_mask_) starts from 40 mJ cm^−2^ to 1211 mJ cm^−2^ with 50 mJ cm^−2^ increments, the dosage on the resist (Dose_resist_) needs to divide Dose_mask_ with tool factors corresponding to various grating pairs. We use the open source SMILE metrology software^30^ to obtain unbiased LWR. The sample for XPS study was directly exposed to EUV light without the mask at the National Synchrotron Radiation Research Center (NSRRC), TLS 21B2.

### Pattern development

The films after e-beam or EUV exposure were baked at 80 °C for 60 s before cooling at room temperature. The pattern was developed with 2-heptanone for 60 s before baking at 90 °C for 90 s.

### High resolution X-ray photoelectron spectroscopy (HRXPS)

HRXPS data were measured on a ULVAC-PHI Quantera II, with a monochromatic Al Kα source (energy of 1486.7 eV). A survey spectrum was obtained with a pass energy of 280 eV and an energy step of 1 eV; a pass energy of 55 eV and energy step of 0.1 eV were employed for O, C, and Sn high-resolution spectra. Thin films were prepared in 25–30 nm thickness and baked at 80 °C for 60 s for EUV exposure at the NSRRC center, Taiwan. After light exposure, standard development was performed with 2-heptanone for 60 s before baking at 90 °C for 90 s. This film is used for HRXPS study.

### FT-IR measurement

A thin film of compound 1 was coated on a 2.5 × 5 cm^2^ silicon wafer by spreading a 4-methyl 2-pentanol solution (2.5 wt%, 0.90 ml) over this wafer substrate. This wafer was dried in air at room temperature for 24 h. The coated film was further baked at 90 °C for 60 s. After EUV exposure, this film was developed with 2-haptanone for 10 s to show the exposed area; the wafer was further baked at 90 °C for 90 s before FT-IR measurement. The operation was performed in air on a Bruker model Tensor 27 equipped with a KBr beam splitter. The signals were collected through transmission mode with an MCT (mercury cadmium telluride) detector; the resolution is 4 cm^−1^.

### Procedure for synthesis of clusters 1 and 2

Butyltin trichloride (0.50 g, 1.77 mmol) and 2-methyl-3-butenoic silver carboxylate (1.09 g, 5.31 mmol) were heated and refluxed in dichloromethane (20 ml) for 8 hours, and the solution was concentrated before recrystallization in a mixed solvent of dichloromethane and *n*-hexane, affording colorless crystals of cluster 1 (345 mg, 0.30 mmol, 59% yield).

Vinyltin trichloride (0.70 g, 2.90 mmol) and 2-methylbutyric silver carboxylate (1.50 g, 7.20 mmol) were heated and refluxed in dichloromethane (15 ml) for 8 h, and the solution was evaporated to dryness and recrystallized in dichloromethane and *n*-hexane, affording colorless crystals of cluster 2 (571 mg, 0.48 mmol, 65% yield). Spectral data are provided in the ESI.†

## Author contribution

The passport name of R.-S. Liu is Jui-Hsiung Liu, who is responsible for all synthetic work. T.-S. Gau and B.-J. Lin took care of the lithographic development. P.-W. Chiu and B.-H. Chen conducted e-beam lithographic work. J. R.-Wu, T.-A. Lin and Y. R. Wu conducted the synthesis and characterization of tin photoresists.

## Conflicts of interest

The authors declare no conflict of interest.

## Supplementary Material

NA-005-D3NA00131H-s001

NA-005-D3NA00131H-s002
