# Supplementary material for: Novel hexameric tin carboxylate clusters as efficient negative-tone EUV photoresists: high resolution with well-defined patterns under low energy doses
Source: Nanoscale Adv. 2023 Apr 28;5(11):3033–43. doi: 10.1039/d3na00131h (PMC10228491; doi:10.1039/d3na00131h)
Supplement: NA-005-D3NA00131H-s001 [file NA-005-D3NA00131H-s001.pdf]

Electronic Supplementary Material (ESI) for Nanoscale Advances.
This journal is © The Royal Society of Chemistry 2023

Supporting Information

Novel Hexameric Tin Carboxylate Clusters as Efficient Negative-Tone EUV Photoresists: High resolution with Well-Defined Patterns using Low Energy Doses

Ting-An Lin[a], Yan-Ru Wu[a], Po-Hsiung Cheng,[b] Tsi-Sheng Gau [b,c] Burn-Jeng Lin*,[b,c]*

Po-Wen Chiu[b,c] and Rai-Shung Liu[a]**

[a]Frontier Research Center for Matter Science and Technology, Department of Chemistry, [b]TSMC-NTHU Joint Research Center, [c]Department of Electric Engineering, National Tsing-Hua University, Hsinchu, Taiwan, ROC---email:rsliu@mx.nthu.edu.tw

Content:

1. SEM images E-beam lithography patterns --- S1

2. SEM images EUV lithography patterns ---S2

3. Table s1: LWR values for cluster 1---S7

4. X-ray crystallographic structures and data ---S7

5. Spectral data of key compounds --S13

6. ^{1}H and ^{13}C NMR of key compounds --S14

1. SEM image of E-beam lithography patterns

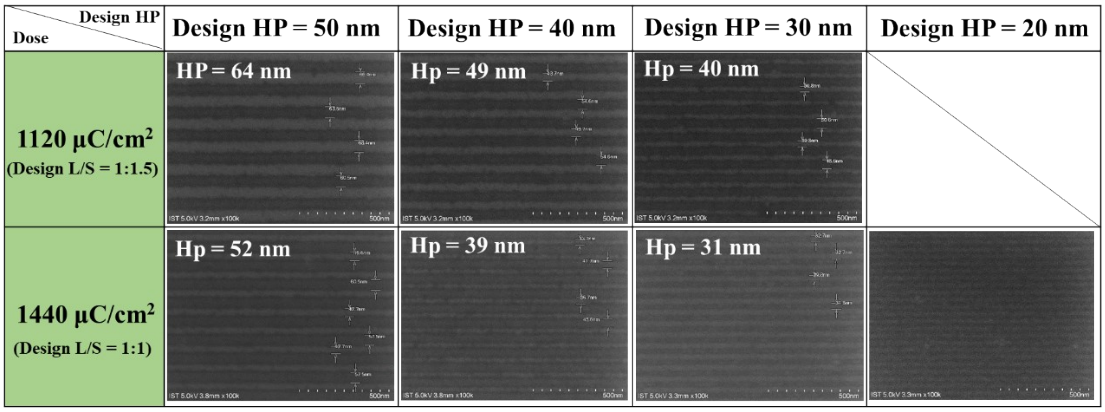

Figure S1. SEM images of E-beam lithography patterns for cluster **1**; Process parameter: 1.5 wt%, THK = 20.9 nm, Developer: 2-Heptanone 60 s, PEB= 80℃ 60 s

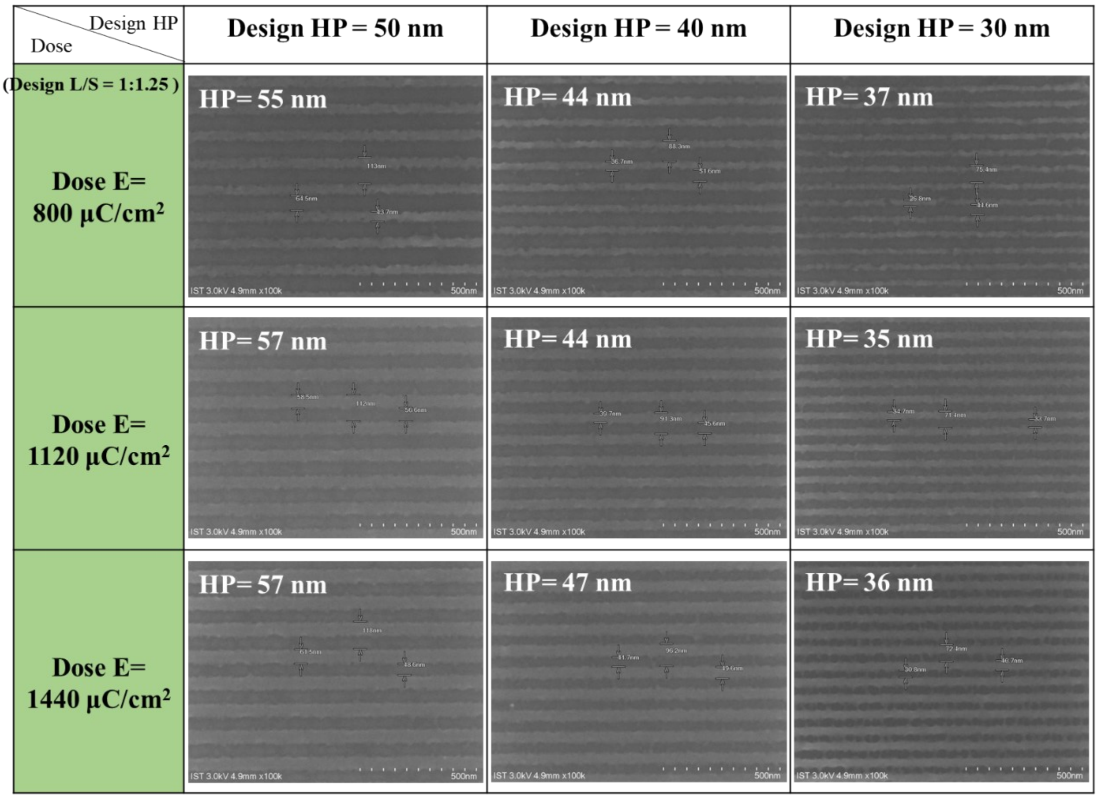

Figure S2. SEM images of E-beam lithography patterns for cluster **2**; Process parameter: 1.75 wt%, THK = 22.9 nm, Developer: 2-Heptanone 60 s, PEB = 80℃ 60 s

2. SEM image of EUV lithography patterns

Design HP= 50 nm	Design HP= 35 nm	Design HP= 25 nm

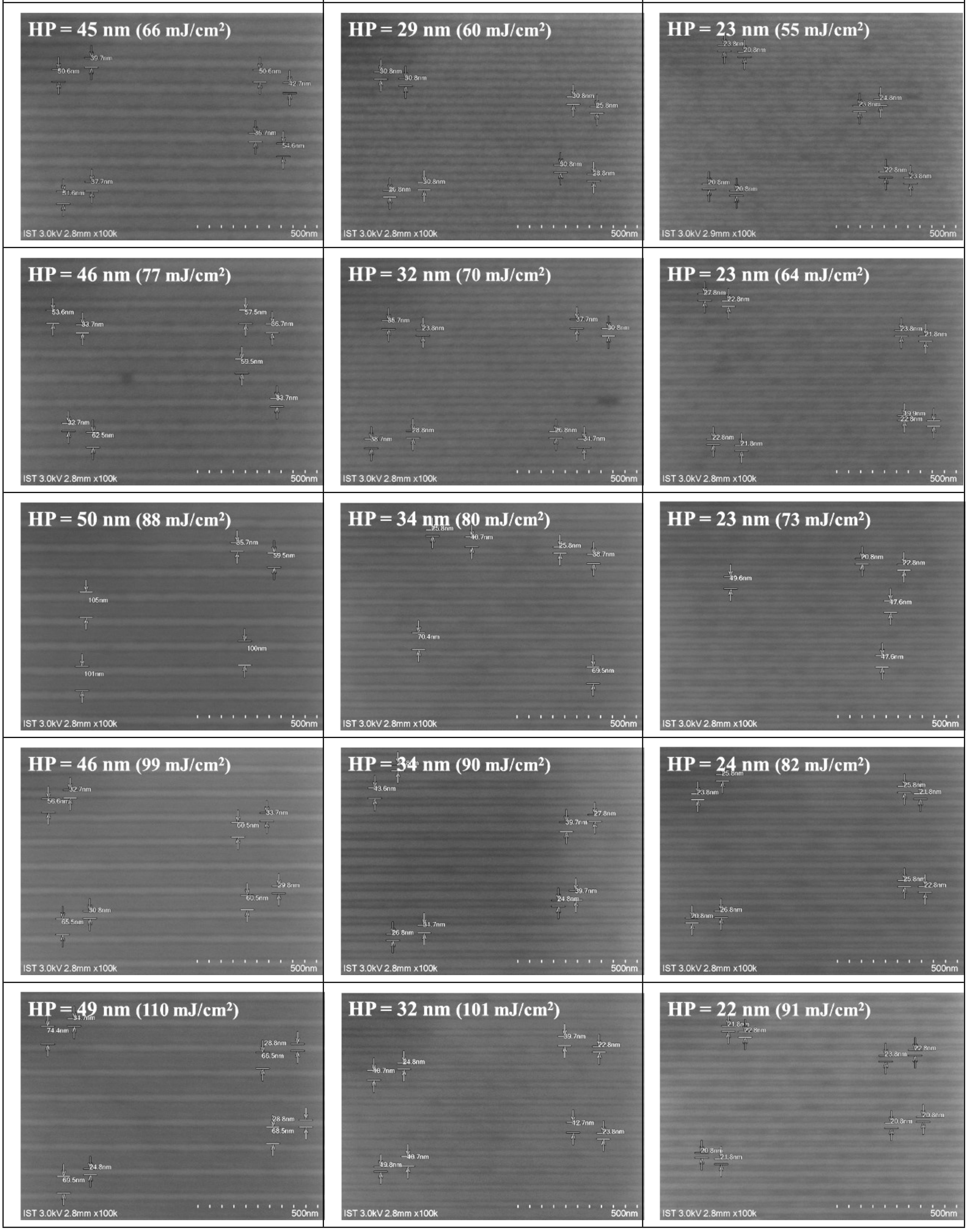

Figure S3. SEM images of EUV lithography patterns for cluster **1:** HP=50, 35, 25 nm at different dose. Process parameter: 1.5 wt%, THK = 20.9 nm, Developer: 2-Heptanone 60 s, PEB= 80℃ 60 s

Design HP= 22 nm	Design HP= 18 nm	Design HP= 16 nm

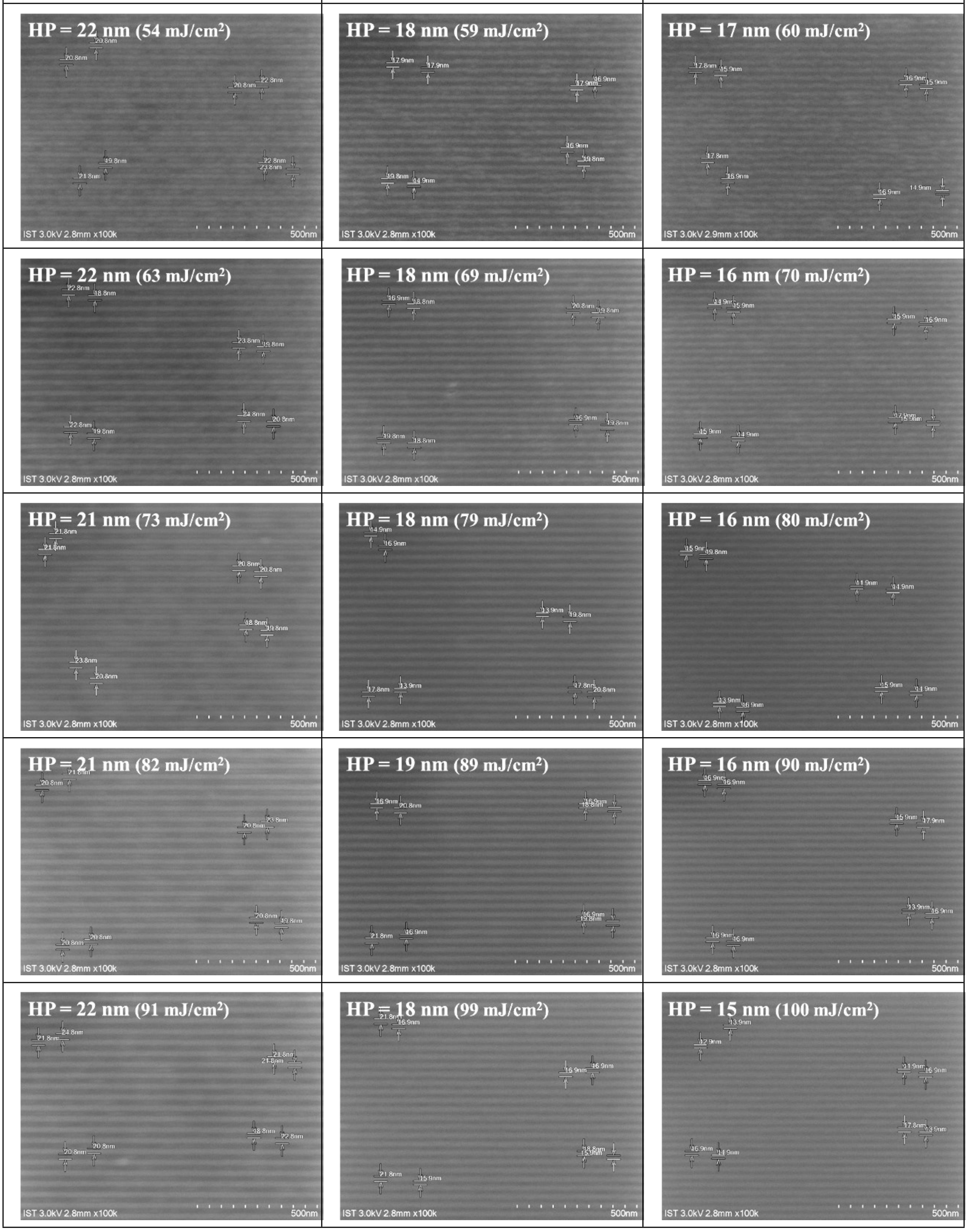

Figure S4. SEM images of EUV lithography patterns for cluster **1:** HP=22, 18, 16 nm at different dose. Process parameter: 1.5 wt%, THK = 20.9 nm, Developer: 2-Heptanone 60 s, PEB= 80℃ 60 s

Design HP= 50 nm	Design HP= 35 nm	Design HP= 25 nm

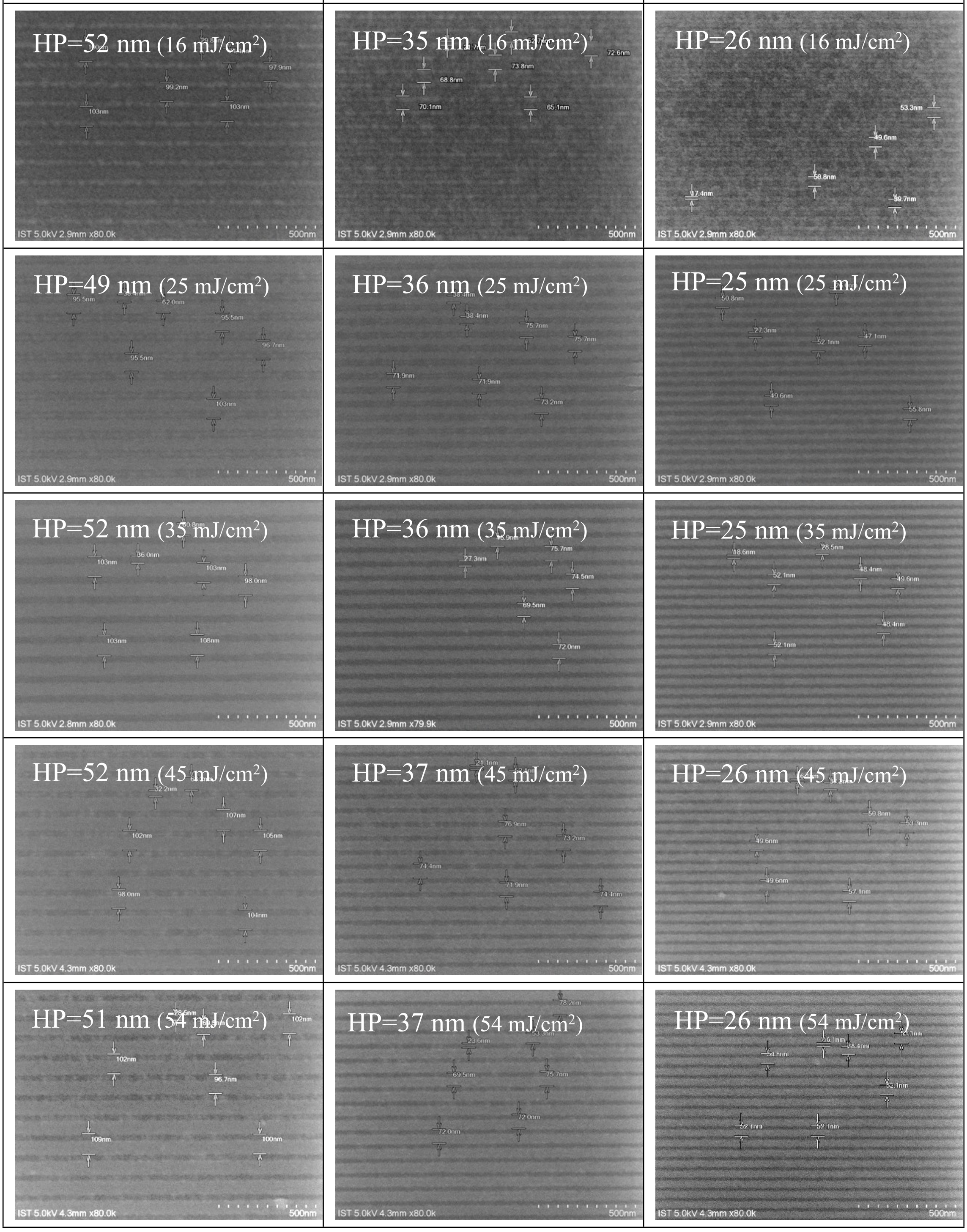

Figure S5. SEM images of EUV lithography patterns for cluster **2**: HP=50, 35, 25 nm at different dose. Process parameter: 1.75 wt%, PAB = 60°C 60s, THK = 22.9 nm, Developer: 2-Heptanone 60 s, No PEB.

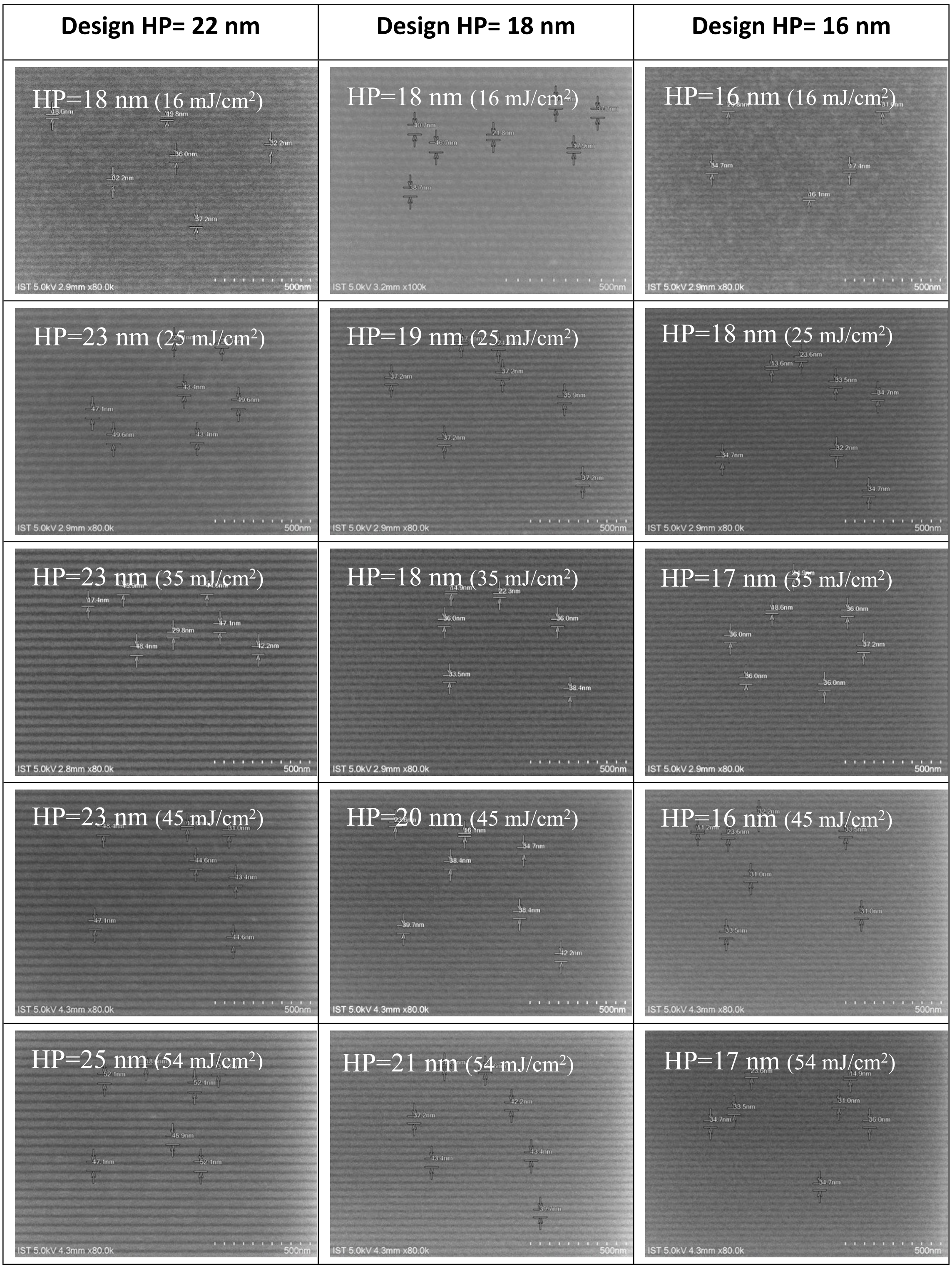

Figure S6. SEM images of EUV lithography patterns for cluster 2. HP=22, 18, 16 nm at different dose. Process parameter: 1.75 wt%, PAB = 60°C 60s, THK = 22.9 nm, Developer: 2-Heptanone 60 s, No PEB.

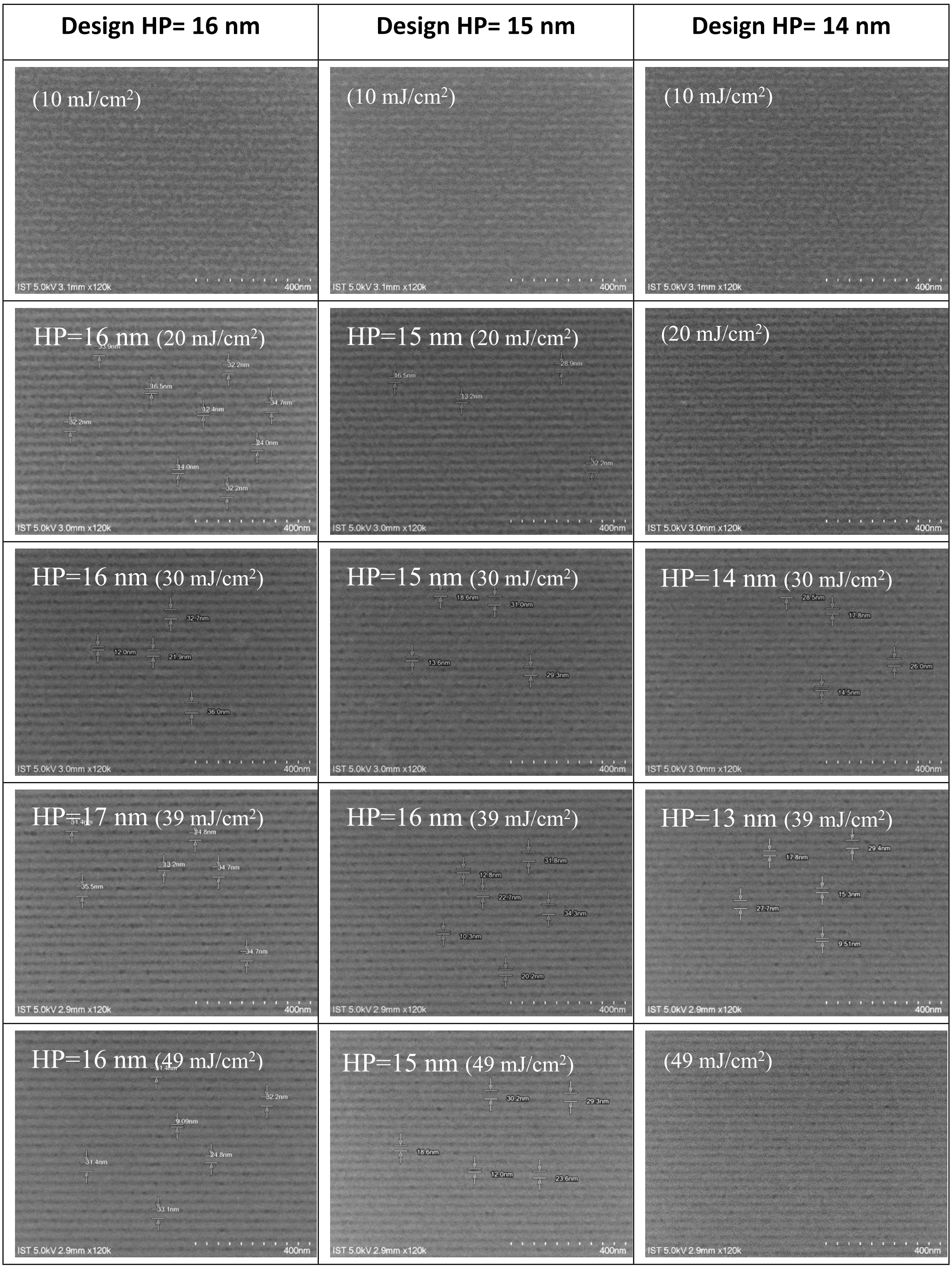

Figure S7. SEM images of EUV lithography patterns for cluster 2, HP=16, 15, 14 nm at different dose. Process parameter: 1.75 wt%, PAB = 60°C 40s, THK = 20.62 nm, Developer: 2-Heptanone 60 s, No PEB.

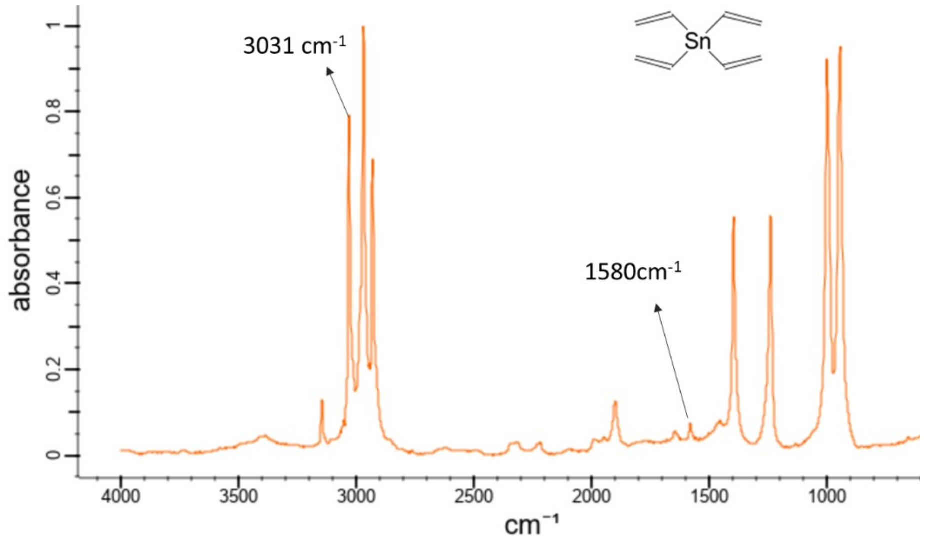

Figure S8. IR spectra of Sn(vinyl)$_4$

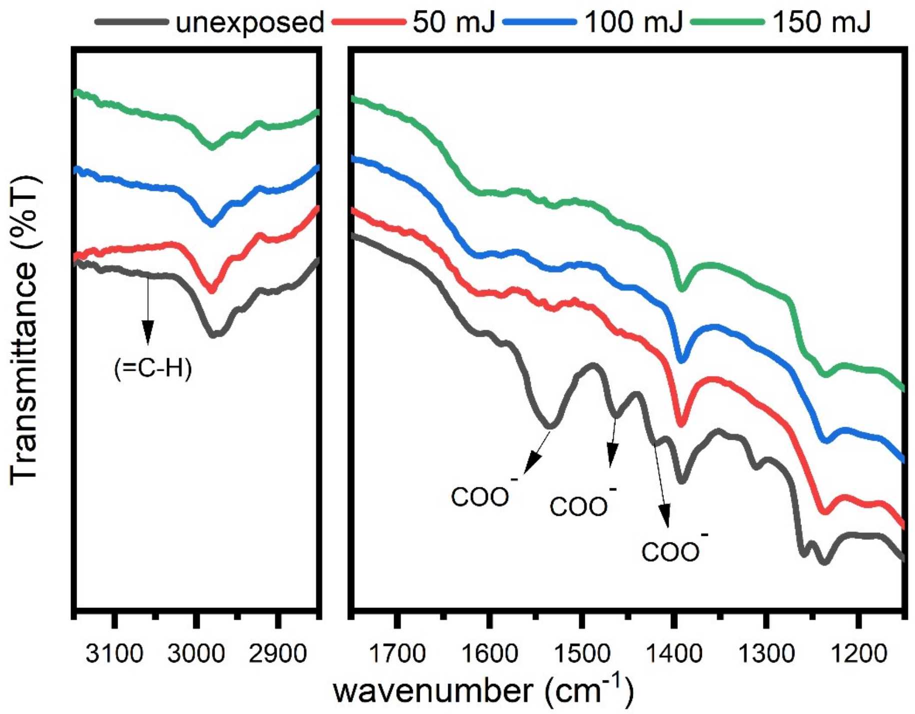

Figure S9. Spectra of photoresist 2 in which the two regions are plotted on equal Magnitudes.

3. **Table S1. LWR values for cluster 1 :**

**Cluster 1 HP = 18 nm**	
Dose (mJ/cm^2)	LWR (nm)
79	4.6
89	4.9
99	5.2

The computation is based on a software: see: l. Mochi, M. Vockenhuber, T. Allenet, Y. Ekinci, *Proc. SPIE*, 2021, **11855**, 1185502

4. X-ray crystallographic structures and data

4.1 Crystal data and X-ray structure of cluster 1 :

1
CCDC (2177714)

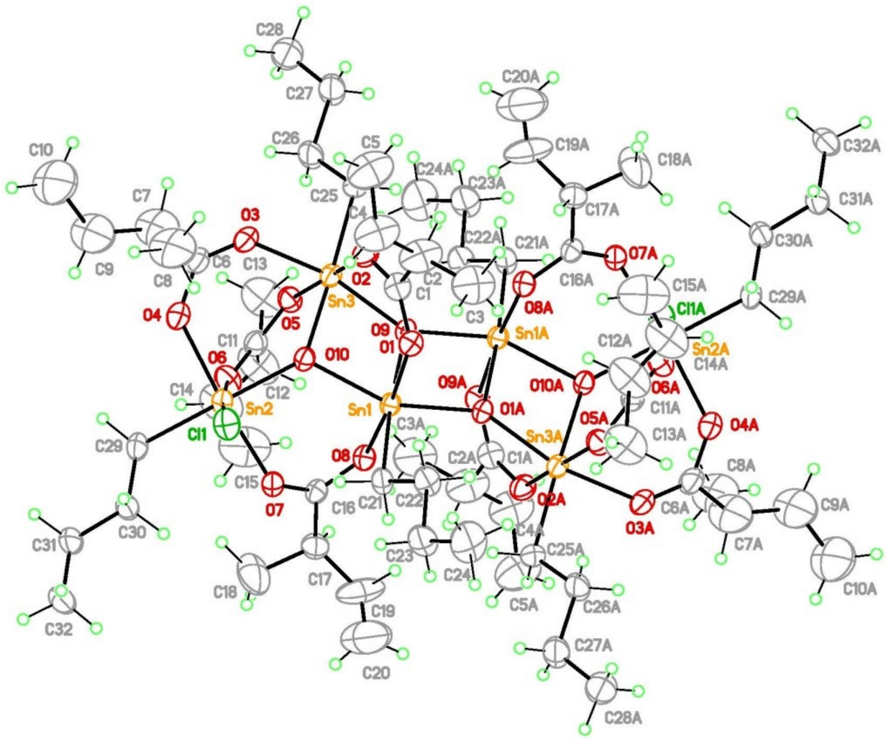

Table 1 Crystal data and structure refinement for 220347lt_auto.

Identification code	220347lt_auto
Empirical formula	$C_{32}H_{55}ClO_{10}Sn_3$
Formula weight	991.28
Temperature/K	99.99(10)
Crystal system	triclinic

Space group	P-1
a/Å	12.4749(4)
b/Å	12.5391(3)
c/Å	15.0700(4)
α/°	90.977(2)
β/°	104.825(3)
γ/°	117.675(3)
Volume/Å^3	1992.54(11)
Z	2
ρ_{calc}g/cm^3	1.652
μ/mm^{-1}	15.858
F(000)	988.0
Crystal size/mm^3	0.21 × 0.14 × 0.06
Radiation	Cu Kα (λ = 1.54184)
2Θ range for data collection/°	6.144 to 145.92
Index ranges	-15 ≤ h ≤ 14, -13 ≤ k ≤ 15, -18 ≤ l ≤ 18
Reflections collected	26569
Independent reflections	7521 [R_{int} = 0.0478, R_{sigma} = 0.0387]
Data/restraints/parameters	7521/317/536
Goodness-of-fit on F^2	1.273
Final R indexes [I>=2σ (I)]	R_1 = 0.0592, wR_2 = 0.1713
Final R indexes [all data]	R_1 = 0.0678, wR_2 = 0.1778
Largest diff. peak/hole / e Å^{-3}	1.63/-1.52

4.3 Crystal data and X-ray structure of cluster 2 :

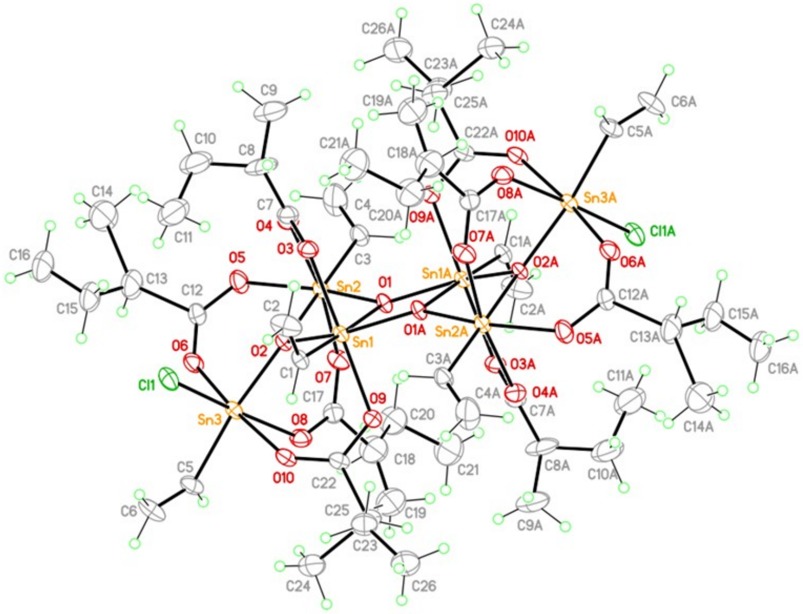

Table 1. Crystal data and structure refinement for 210753lt_0m_a.

Identification code	210753lt_0m_a	
Empirical formula	C52 H90 Cl2 O20 Sn6	
Formula weight	1818.27	
Temperature	100(2) K	
Wavelength	0.71073 Å	
Crystal system	Triclinic	
Space group	P-1	
Unit cell dimensions	a = 10.7499(9) Å	α= 67.810(4)°.
	b = 13.2738(11) Å	β= 71.148(4)°.
	c = 14.0693(11) Å	γ = 73.030(4)°.
Volume	1725.8(3) Å^3	
Z	1	
Density (calculated)	1.750 Mg/m^3	
Absorption coefficient	2.278 mm^{-1}	
F(000)	896	
Crystal size	0.23 x 0.20 x 0.06 mm^3	
Theta range for data collection	1.610 to 26.514°.	
Index ranges	-13<=h<=12, -16<=k<=16, -17<=l<=16	
Reflections collected	28957	
Independent reflections	7053 [R(int) = 0.0502]	
Completeness to theta = 25.242°	99.6 %	

Absorption correction	Semi-empirical from equivalents
Max. and min. transmission	0.7454 and 0.6419
Refinement method	Full-matrix least-squares on F^2
Data / restraints / parameters	7053 / 414 / 525
Goodness-of-fit on F^2	1.080
Final R indices [I>2sigma(I)]	R1 = 0.0406, wR2 = 0.1111
R indices (all data)	R1 = 0.0539, wR2 = 0.1288
Extinction coefficient	n/a
Largest diff. peak and hole	1.401 and -1.066 e.Å^{-3}

5. Spectral data of key compounds

5.1 Spectral data for cluster 1 :

Transparent crystal (59%) ; ^1H NMR (400 MHz, $CDCl_3$) : δ 5.92-5.88 (m, 8H), 5.12-5.07 (m, 16H), 3.12-3.04 (m, 8H), 1.73-1.62 (m, 24H), 1.34-1.32 (m, 12H), 1.24-1.18 (m, 24H), 0.88 (d, J = 7.1 Hz, 18H)； ^{13}C NMR (125 MHz, $CDCl_3$) : δ 182.7, 181.8, 138.7, 137.9, 137.7, 115.8, 115.4, 115.1, 114.9, 114.5, 114.4, 46.5, 46.5, 46.3, 46.2, 35.6, 35.5, 27.7, 27.5, 27.2, 27.1, 26.7, 26.4, 26.2, 26.1, 25.7, 17.2, 17.0； ^{119}Sn NMR (186 MHz, $CDCl_3$) : δ -483.9, -522.3, -523.6, -551.1； HRMS (ESI+, m/z) Calcd. For $^{12}C_{64}{}^{1}H_{110}{}^{35}Cl_2{}^{16}O_{20}{}^{120}Sn_6$ $[M+H]^+$: 1989.11, found : 1989.87; EA Anal. Calcd. for $C_{64}H_{110}Cl_2O_{20}Sn_6$: C : 38.77% ; H : 5.59%, found : C : 38.65% ; H : 5.58%.

5.2 Spectral data for cluster 2 :

Transparent crystal (65%)； ^1H NMR（500 MHz, $CDCl_3$）： δ 6.22-5.91（m, 18H）, 2.44-2.24（m, 8H）, 1.72-1.35（m, 16H）, 1.17-1.07（m, 24H）, 0.93-0.85（m, 24H）； ^{13}C NMR（125 MHz, $CDCl_3$）： δ 186.0, 185.4, 185.2, 185.1, 184.6, 184.1, 183.5, 145.2, 141.9, 141.1, 140.8, 139.7, 139.3, 138.7, 138.3, 138.1, 136.3, 135.5, 135.1, 134.5, 133.9, 133.5, 133.0, 132.1, 131.8, 131.2, 123.0, 43.9, 43.8, 43.8, 43.6, 40.5, 27.3, 27.2, 27.1, 26.8, 16.9, 16.8, 16.7, 16.5, 11.7, 11.5； ^{119}Sn NMR（186 MHz, $CDCl_3$）： δ -183.96, -539.25, -542.63, -547.88. HRMS（ESI+, m/z）Calcd. For $C_{52}H_{90}Cl_2O_{20}Sn_6$ $[M+H]^+$： 1823.95, found： 1824.75； EA Anal. Calcd. for $C_{52}H_{90}Cl_2O_{20}Sn_6$： C： 34.35%； H： 4.99%, found： C： 34.32%； H： 4.94%.

6. ^{1}H and ^{13}C NMR of key compounds

6.1 Spectral data for cluster 1

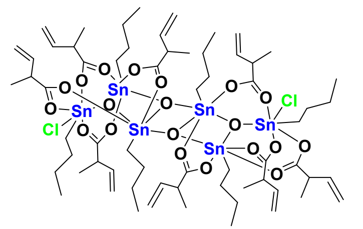

1

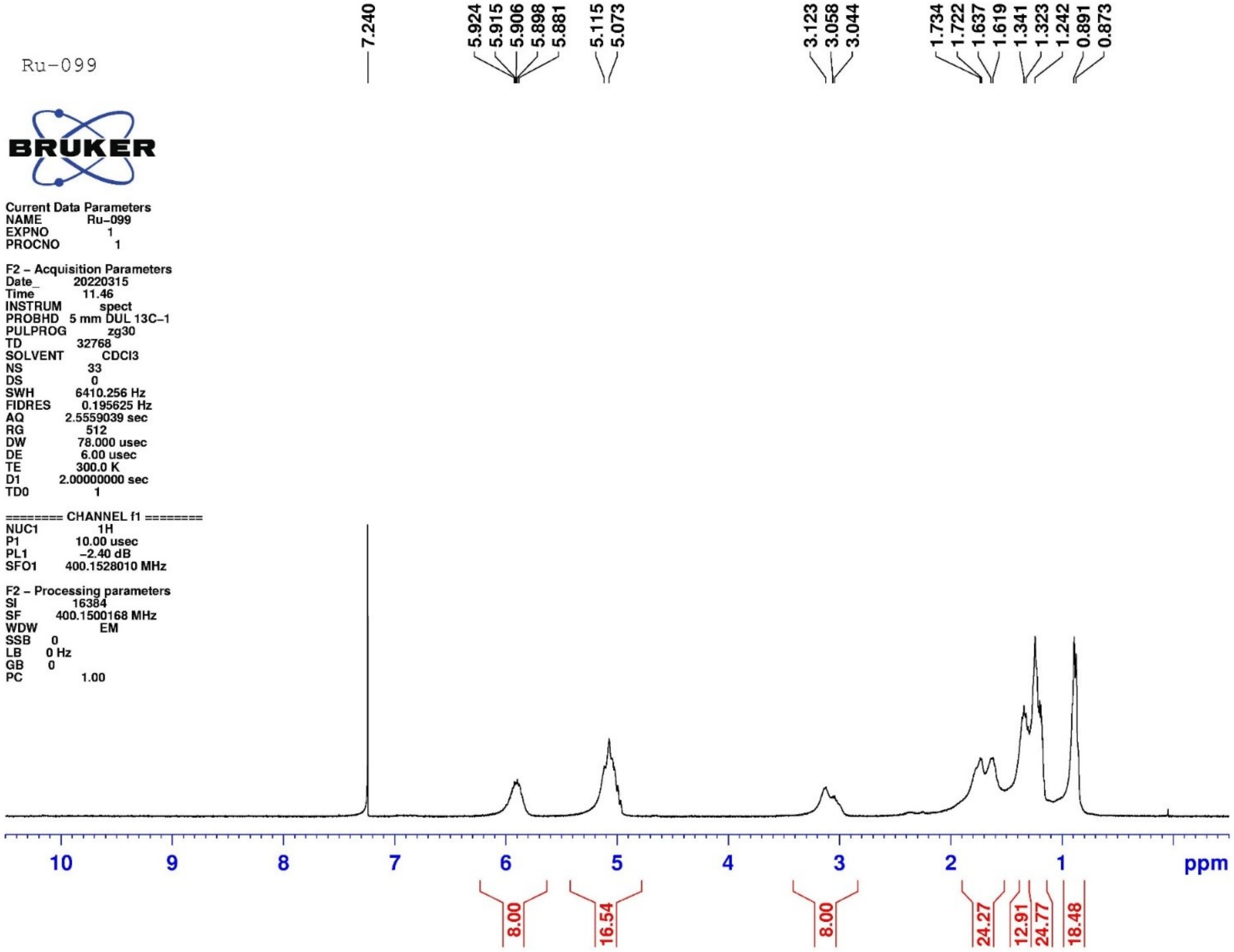

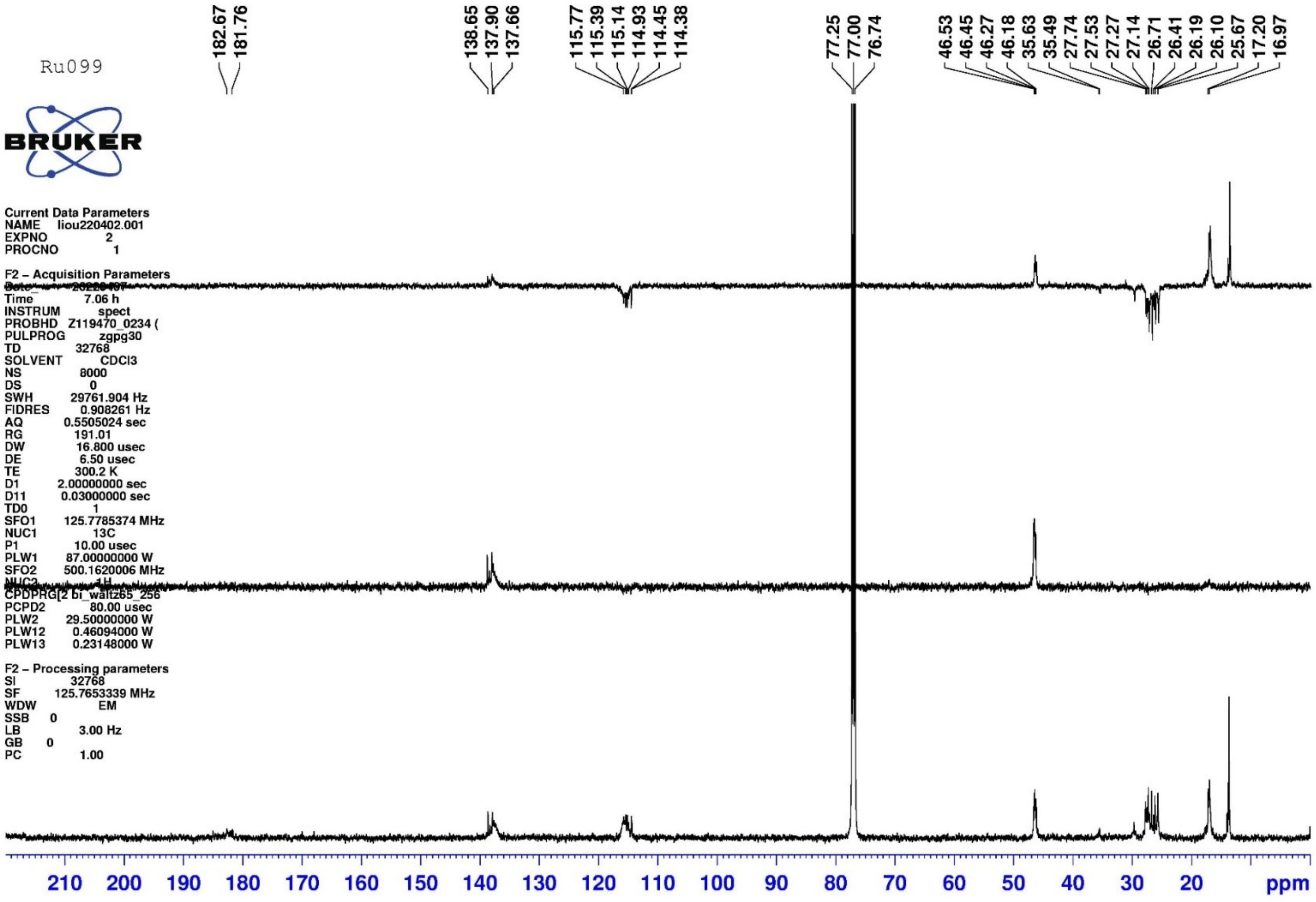
Ru099
182.67
181.76
138.65
137.90
137.66
115.77
115.39
115.14
114.93
114.45
114.38
77.25
77.00
76.74
46.53
46.45
46.27
46.18
35.63
35.49
27.74
27.53
27.27
27.14
26.71
26.41
26.19
26.10
25.67
17.20
16.97
Current Data Parameters
NAME liou220402.001
EXPNO 2
PROCNO 1
F2 - Acquisition Parameters
Time 7.06 h
INSTRUM spect
PROBHD Z119470_0234 (
PULPROG zgpg30
TD 32768
SOLVENT CDCl3
NS 8000
DS 0
SWH 29761.904 Hz
FIDRES 0.908261 Hz
AQ 0.5505024 sec
RG 191.01
DW 16.800 usec
DE 6.50 usec
TE 300.2 K
D1 2.00000000 sec
D11 0.03000000 sec
TD0 1
SFO1 125.7785374 MHz
NUC1 13C
P1 10.00 usec
PLW1 87.00000000 W
SFO2 500.1620006 MHz
CPDPRG[2 bi_waltz65_256
PCPD2 80.00 usec
PLW2 29.50000000 W
PLW12 0.46094000 W
PLW13 0.23148000 W
F2 - Processing parameters
SI 32768
SF 125.7653339 MHz
WDW EM
SSB 0
LB 3.00 Hz
GB 0
PC 1.00
210 200 190 180 170 160 150 140 130 120 110 100 90 80 70 60 50 40 30 20 ppm

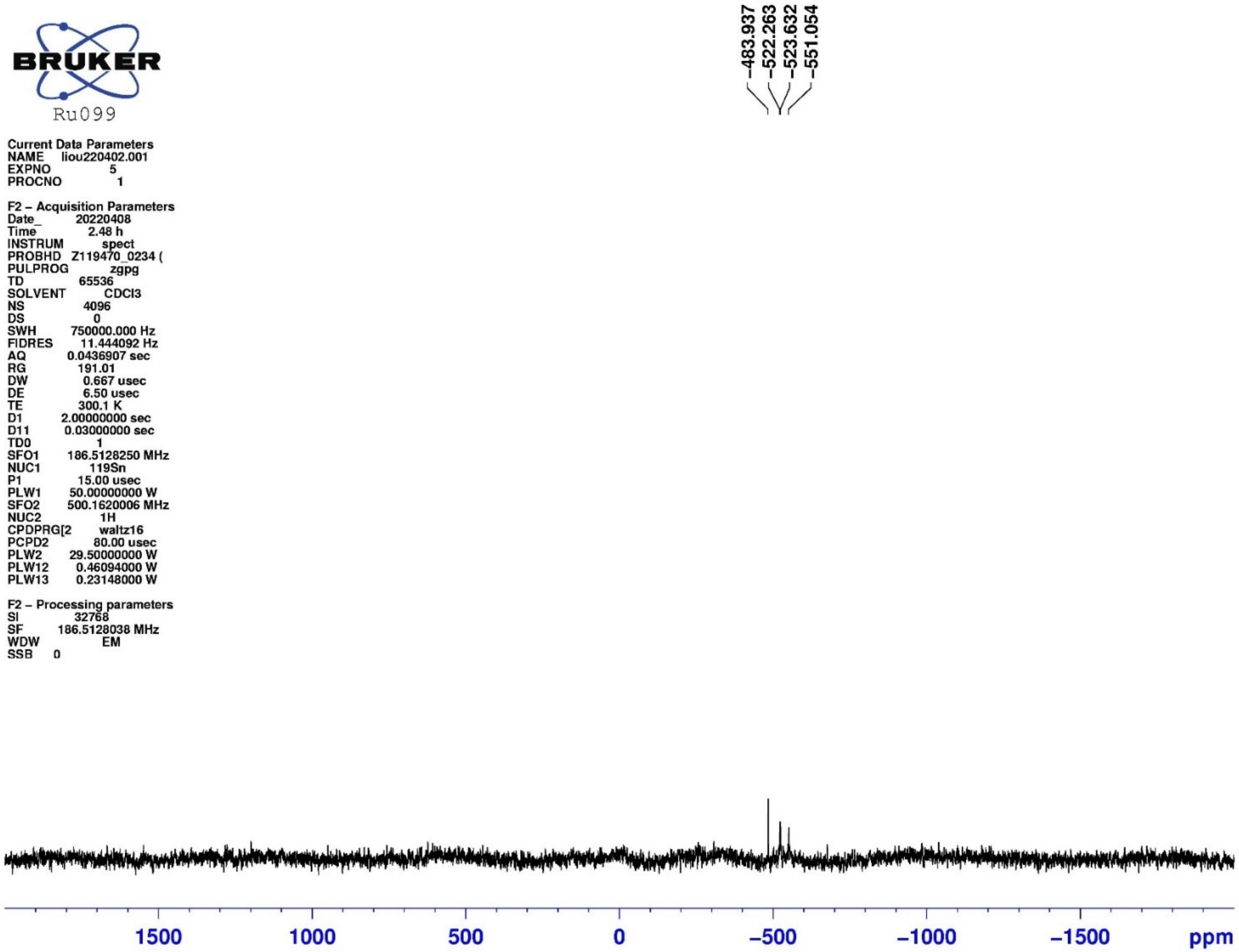
BRUKER
Ru099
Current Data Parameters
NAME liou220402.001
EXPNO 5
PROCNO 1
F2 – Acquisition Parameters
Date_ 20220408
Time 2.48 h
INSTRUM spect
PROBHD Z119470_0234 (
PULPROG zgpg
TD 65536
SOLVENT CDCl3
NS 4096
DS 0
SWH 750000.000 Hz
FIDRES 11.444092 Hz
AQ 0.0436907 sec
RG 191.01
DW 0.667 usec
DE 6.50 usec
TE 300.1 K
D1 2.00000000 sec
D11 0.03000000 sec
TD0 1
SFO1 186.5128250 MHz
NUC1 119Sn
P1 15.00 usec
PLW1 50.00000000 W
SFO2 500.1620006 MHz
NUC2 1H
CPDPRG[2 waltz16
PCPD2 80.00 usec
PLW2 29.50000000 W
PLW12 0.46094000 W
PLW13 0.23148000 W
F2 – Processing parameters
SI 32768
SF 186.5128038 MHz
WDW EM
SSB 0
–483.937
–522.263
–523.632
–551.054
1500
1000
500
0
–500
–1000
–1500
ppm

6.2 Spectral data for cluster 2

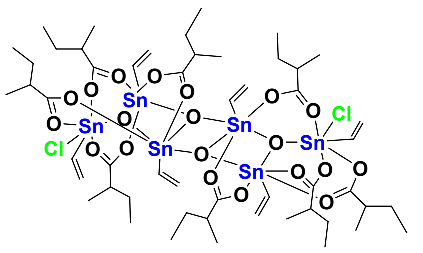

2

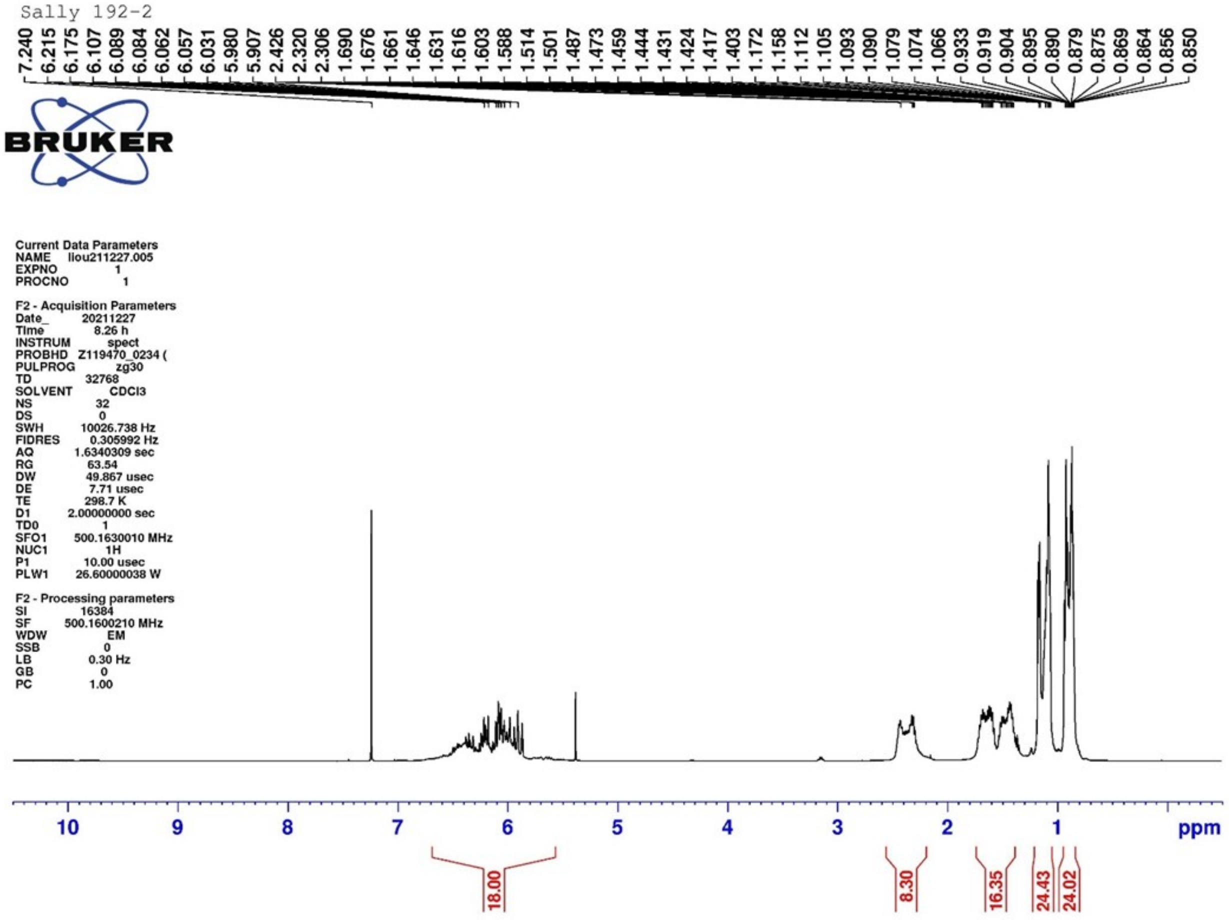

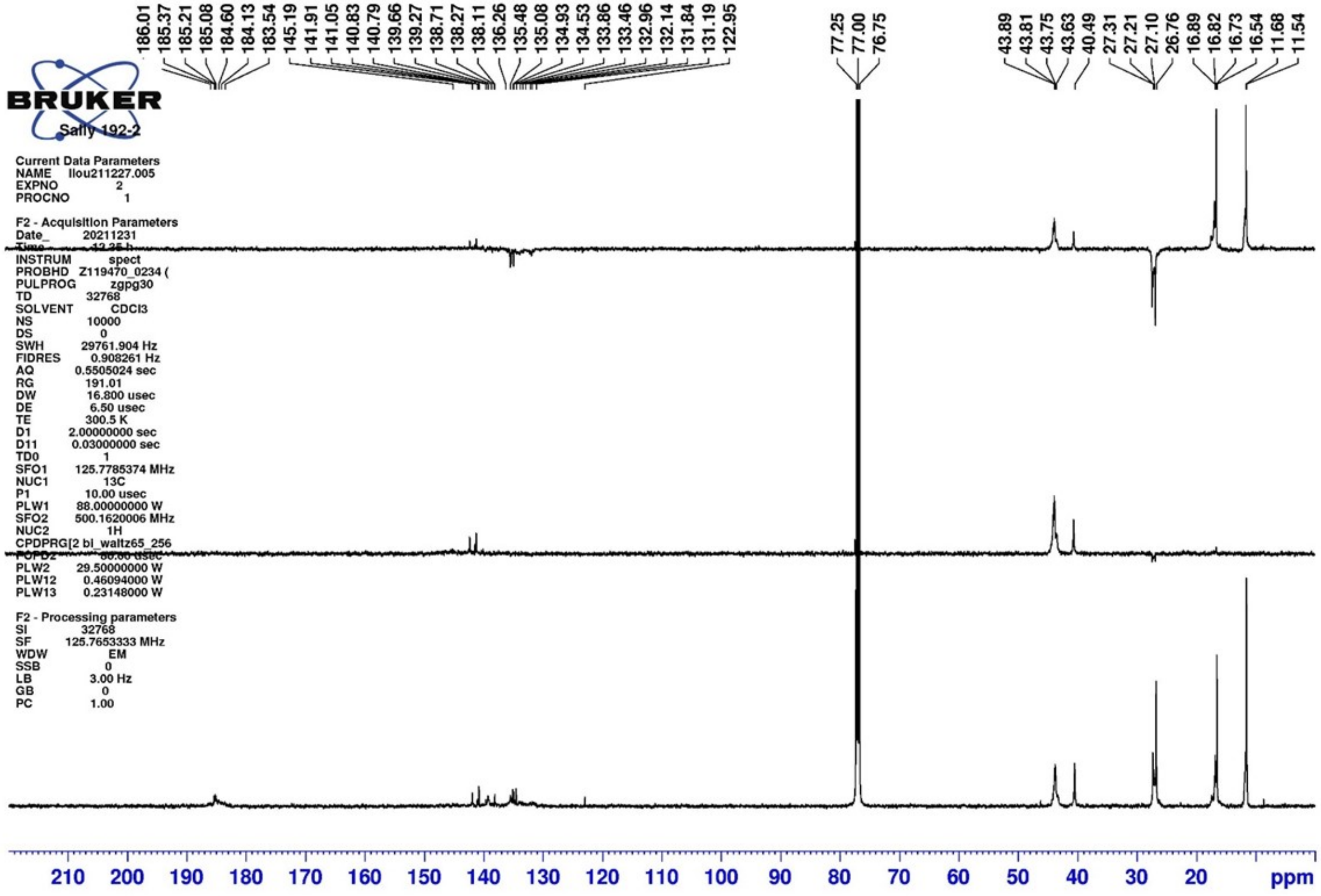
186.01
185.37
185.21
185.08
184.60
184.13
183.54
145.19
141.91
141.05
140.83
140.79
139.66
139.27
138.71
138.27
138.11
136.26
135.48
135.08
134.93
134.53
133.86
133.46
132.96
132.14
131.84
131.19
122.95
77.25
77.00
76.75
43.89
43.81
43.75
43.63
40.49
27.31
27.21
27.10
26.76
16.89
16.82
16.73
16.54
11.68
11.54
BRUKER
Sally 192-2
Current Data Parameters
NAME llou211227.005
EXPNO 2
PROCNO 1
F2 - Acquisition Parameters
Date_ 20211231
INSTRUM spect
PROBHD Z119470_0234 (
PULPROG zgpg30
TD 32768
SOLVENT CDCl3
NS 10000
DS 0
SWH 29761.904 Hz
FIDRES 0.908261 Hz
AQ 0.5505024 sec
RG 191.01
DW 16.800 usec
DE 6.50 usec
TE 300.5 K
D1 2.00000000 sec
D11 0.03000000 sec
TD0 1
SFO1 125.7785374 MHz
NUC1 13C
P1 10.00 usec
PLW1 88.00000000 W
SFO2 500.1620006 MHz
NUC2 1H
CPDPRG[2 bi_waltz65_256
PLW2 29.50000000 W
PLW12 0.46094000 W
PLW13 0.23148000 W
F2 - Processing parameters
SI 32768
SF 125.7653333 MHz
WDW EM
SSB 0
LB 3.00 Hz
GB 0
PC 1.00
210
200
190
180
170
160
150
140
130
120
110
100
90
80
70
60
50
40
30
20
ppm

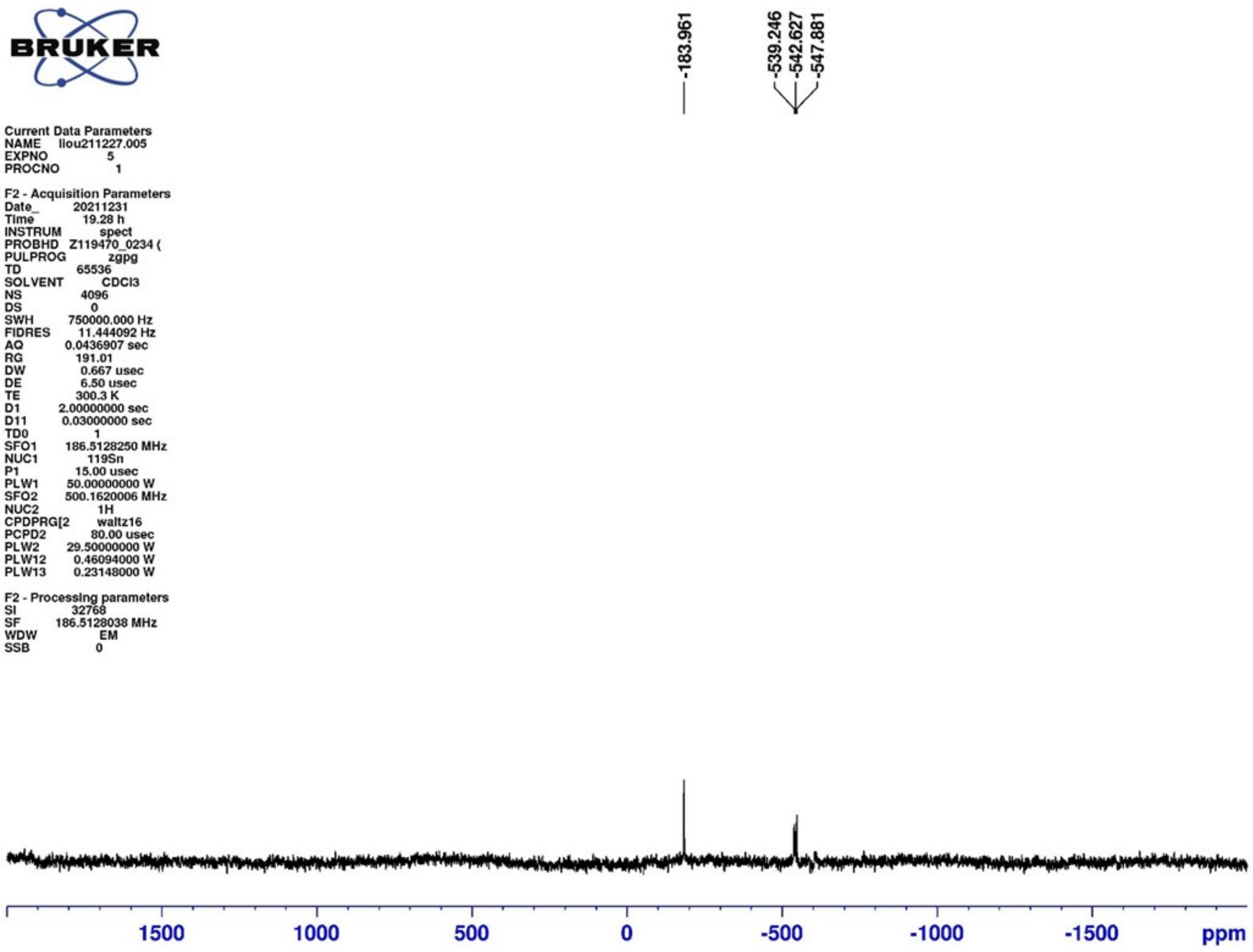
BRUKER
-183.961
-539.246
-542.627
-547.881
Current Data Parameters
NAME liou211227.005
EXPNO 5
PROCNO 1
F2 - Acquisition Parameters
Date_ 20211231
Time 19.28 h
INSTRUM spect
PROBHD Z119470_0234 (
PULPROG zgpg
TD 65536
SOLVENT CDCl3
NS 4096
DS 0
SWH 750000.000 Hz
FIDRES 11.444092 Hz
AQ 0.0436907 sec
RG 191.01
DW 0.667 usec
DE 6.50 usec
TE 300.3 K
D1 2.00000000 sec
D11 0.03000000 sec
TD0 1
SFO1 186.5128250 MHz
NUC1 119Sn
P1 15.00 usec
PLW1 50.00000000 W
SFO2 500.1620006 MHz
NUC2 1H
CPDPRG[2 waltz16
PCPD2 80.00 usec
PLW2 29.50000000 W
PLW12 0.46094000 W
PLW13 0.23148000 W
F2 - Processing parameters
SI 32768
SF 186.5128038 MHz
WDW EM
SSB 0
1500
1000
500
0
-500
-1000
-1500
ppm
